# Lifetimes and spatio-temporal response of protein crystals in intense X-ray microbeams

**DOI:** 10.1107/S2052252517013495

**Published:** 2017-10-13

**Authors:** Matthew A. Warkentin, Hakan Atakisi, Jesse B. Hopkins, Donald Walko, Robert E. Thorne

**Affiliations:** a Physics Department, Cornell University, Clark Hall, Ithaca, NY 14853, USA; b Rubota Corporation, 1260 NW Naito Parkway #609, Portland, OR 97209, USA; c Physics Department, Cornell University, Ithaca, NY 14853, USA; d Cornell High Energy Synchrotron Source, Ithaca, NY 14853, USA; e Advanced Photon Source, Argonne National Laboratory, 9700 South Cass Avenue, Argonne, IL 60439, USA

**Keywords:** protein crystallography, radiation damage, serial crystallography, microcrystallography, structure determination, protein structure, X-ray crystallography, structural biology, intense X-ray microbeams

## Abstract

Serial synchrotron-based crystallography using intense microfocused X-ray beams, fast-framing detectors and protein microcrystals held at 300 K promises to expand the range of accessible structural targets and to increase overall structure-pipeline throughputs. To explore the nature and consequences of X-ray radiation damage under microbeam illumination, the time-, dose- and temperature-dependent evolution of crystal diffraction have been measured with maximum dose rates of 50 MGy s^−1^. At all temperatures and dose rates, the integrated diffraction intensity for a fixed crystal orientation shows non-exponential decays with dose. Non-exponential decays are a consequence of non-uniform illumination and the resulting spatial evolution of diffracted intensity within the illuminated crystal volume. To quantify radiation-damage lifetimes and the damage state of diffracting crystal regions, a revised diffraction-weighted dose (DWD) is defined and it is shown that for Gaussian beams the DWD becomes nearly independent of actual dose at large doses. An apparent delayed onset of radiation damage seen in some intensity–dose curves is in fact a consequence of damage. Intensity fluctuations at high dose rates may arise from the impulsive release of gaseous damage products. Accounting for these effects, data collection at the highest dose rates increases crystal radiation lifetimes near 300 K (but not at 100 K) by a factor of ∼1.5–2 compared with those observed at conventional dose rates. Improved quantification and modeling of the complex spatio-temporal evolution of protein microcrystal diffraction in intense microbeams will enable more efficient data collection, and will be essential in improving the accuracy of structure factors and structural models.

## Abstract 2

The complex evolution of diffracted intensities from protein crystals during irradiation by intense Gaussian X-ray microbeams is measured and analysed. The analysis explains non-exponential intensity decays without invoking sequential damage models, yields a revised metric to quantify the damage state of the crystal after a given irradiation time, explains previous observations of a damage ‘lag’ phase and shows how ultra-intense X-ray microbeams allow the data collected per crystal at and near room temperature to be increased.

## 1. Introduction   

The overwhelming majority of biomacromolecular structures have been and will continue to be determined by X-ray crystallography. Increasing synchrotron-source brilliance allows X-ray flux to be concentrated into smaller and smaller beams. This in turn allows data collection from smaller and smaller crystals, and longstanding challenges in growing large crystals of important targets (*e.g.* membrane proteins and large complexes) to be bypassed. Motivated by the success of serial femtosecond crystallography using X-ray free-electron lasers (XFELs), methods for synchrotron-based serial microcrystallography are being developed (Roessler *et al.*, 2013 ▸; Stellato *et al.*, 2014 ▸; Heymann *et al.*, 2014 ▸; Coquelle *et al.*, 2015 ▸; Botha *et al.*, 2015 ▸; Mueller *et al.*, 2015 ▸; Lyubimov *et al.*, 2015 ▸), combining ultra-intense microfocused beams, multiple crystal holders or continuous crystal-feed systems, and fast-framing detectors with computational tools for indexing, merging and modeling diffraction data from large numbers of crystals (Kabsch, 2010 ▸; Gildea *et al.*, 2014 ▸; Ayyer *et al.*, 2015 ▸; White *et al.*, 2016 ▸; Ginn *et al.*, 2016 ▸). To maximize crystal lifetimes, nearly all protein crystallography is performed on crystals cooled to *T* = 100 K. However, at 300 K crystals have much smaller mosaicities and are more likely to be isomorphous (Kriminski *et al.*, 2002 ▸; Malkin & Thorne, 2004 ▸; Pflugrath, 2004 ▸; Juers & Matthews, 2004 ▸; Farley *et al.*, 2014 ▸). Recently developed computational methods (van den Bedem *et al.*, 2009 ▸; Lang *et al.*, 2010 ▸; Fenwick *et al.*, 2014 ▸) for analysing low-density features in electron-density maps and experimental protocols for 300 K and variable-temperature data collection (Warkentin & Thorne, 2010*b* ▸) are beginning to reveal a wealth of information about the conformational ensemble of a protein that is corrupted on cooling to cryogenic temperatures (Fraser *et al.*, 2009 ▸; Keedy *et al.*, 2015 ▸).

These trends have made understanding, minimizing and modeling damage caused by the illuminating X-rays increasingly important. Inelastic X-ray photon scattering and photoelectron ejection lead to a cascade of chemical and structural processes, from generation of secondary electrons and free radicals, reduction of metal centers, breakage of chemical bonds and formation of hydrogen gas to conformational relaxations and local unfolding, molecular displacements and lattice distortions, and plastic failure (Holton, 2009 ▸; Garman, 2010 ▸; Warkentin *et al.*, 2013 ▸). These processes change the average structure of the protein within the crystal, introduce molecule-to-molecule deviations from the average structure and cause spatial variations in the local lattice spacing and orientation. In X-ray diffraction, these changes manifest as changes in the intensities, shapes and positions of the Bragg peaks with dose, especially at large scattering angles, and in increased diffuse scattering.

Previous radiation-damage studies on protein crystals using large (50–400 µm) X-ray beams and large crystals have established that damage depends on dose (measured in Grays; Gy; 1 Gy = 1 J kg^−1^), determined by the incident photon energy and flux density and the crystal composition, that the integrated intensity in Bragg peaks decays approximately exponentially with dose (Holton, 2009 ▸; Garman, 2010 ▸), and that the rate of radiation damage increases by a factor of ∼30–50 between 100 and 300 K, with substantial protein-to-protein variation (Warkentin *et al.*, 2014 ▸).

Less is known about the response of protein crystals to the intense X-ray microbeams generated using synchrotrons (Smith *et al.*, 2012 ▸). These beams typically have Gaussian intensity profiles with widths of a few to a few tens of micrometres and generate spatially non-uniform damage. At higher X-ray energies, photoelectrons can deposit their energy micrometres from the absorption site (Nave & Hill, 2005 ▸; Sanishvili *et al.*, 2011 ▸; Finfrock *et al.*, 2013 ▸), so that beam and damage profiles may differ. Intense beams degrade diffraction rapidly, so data must be collected using fast-framing detectors, and time-dependent as well as dose-dependent effects may manifest (Warkentin *et al.*, 2012 ▸, 2013 ▸; Owen *et al.*, 2012 ▸, 2014 ▸). Consequently, standard assumptions about the effects of irradiation and induced disorder on diffracted intensities may become invalid, additional errors may be introduced into measured structure factors, and the relatively poor *R* factors of refined protein structures (Holton *et al.*, 2014 ▸) may become even worse. Here, we explore the complex evolution of diffracted intensities from protein crystals illuminated by intense X-ray microbeams and show how these can be interpreted in terms of the spatio-temporal evolution of radiation damage. These results have important consequences for optimizing data collection, for extracting structure factors from measured intensities and for studying the underlying mechanisms of radiation damage.

## 2. Methods   

Data-collection protocols are critical to interpreting the results of radiation-damage studies, so we first give a brief overview of our methods, which are described in more detail below and in the Supporting Information. To assess damage by X-ray microbeams, a large and highly redundant data set of ∼50 000 diffraction frames was acquired from ∼1300 independent positions on 26 crystals of lysozyme and thaumatin at temperatures of 100, 260 and 300 K. Crystals of roughly 100–600 µm in size were illuminated with a Gaussian profile X-ray microbeam (Supplementary Fig. S1) with a full-width at half-maximum (FWHM) of 2.4 × 5.1 µm and delivering peak (at the beam center) and nominal (average within the FWHM) dose rates of ∼49 and ∼35 MGy s^−1^, respectively, a factor of ∼10^3^ larger than in conventional crystallographic practice. To ascertain the dose-rate dependence of the damage, attenuators were used to reduce the dose rate by factors of up to ∼10^3^. Each crystal was held in a fixed orientation and diffraction frames *versus* dose at different dose rates were collected from an array of positions (Supplementary Fig. S2). Bragg peak intensities in each frame were integrated, and this integrated intensity was plotted *versus* dose to yield an intensity–dose relation or ‘dose curve’ at each position and dose rate.

### 2.1. Sample preparation   

Tetragonal thaumatin and tetragonal lysozyme crystals were grown by hanging-drop vapor diffusion using standard recipes (Supporting Information §S1). Crystals for measurements at 260 and 300 K were transferred to a high-viscosity oil to remove external solvent and then harvested and mounted for data collection using microfabricated polyimide or nylon cryoloops. The oil thickness was typically 50–100 µm and was sufficient to prevent any dehydration (as monitored through unit-cell parameters) during the ∼15–30 min that each crystal was examined. As an additional check, some oil-covered crystals were also mounted in polyimide capillaries and gave similar results. Crystals for measurements at 100 K were first soaked in a 20%(*w*/*v*) glycerol solution and were then transferred to a drop of low-viscosity oil to remove external solvent before mounting and insertion into a cold nitrogen-gas stream.

### 2.2. Beamline setup and beam characteristics   

X-ray diffraction data were collected at the D-hutch of Argonne X-ray Science Division beamline 7-ID at the Advanced Photon Source. A 10 keV X-ray beam was focused using two Kirkpatrick–Baez mirrors onto a fluorescent screen at the sample position. The beam profile was measured by scanning the edge of a gallium arsenide wafer in *x*, *y* and *z*, measuring the transmitted intensity and fitting the resulting curve with an error function to extract the FWHM. An example beam image and profile determined in this way are shown in Supplementary Fig. S1. The beam position and size were continuously monitored and were exceptionally stable, requiring only minor adjustments. The measured beam FWHM values were 2.5 µm (vertical) by 5.1 µm (horizontal), and the divergence of the focused beam at the sample was approximately 0.07° (vertical) by 0.13° (horizontal). The incident flux was monitored with a calibrated 7 cm ion chamber located 0.64 m downstream of the sample. Corrected for air absorption, the flux at the sample position was 1.2 × 10^12^ photons s^−1^.

### 2.3. Dose-rate calculations   

Using the measured photon flux and FWHM, the peak flux density within the FWHM was ∼1.2 × 10^17^ photons mm^−2^ s^−1^. The corresponding nominal (average within the FWHM) and peak dose rates calculated using *RADDOSE*-3*D* (Zeldin, Gerstel *et al.*, 2013 ▸) were 36 and 49 MGy s^−1^, respectively, for thaumatin and 33 and 45 MGy s^−1^, respectively, for lysozyme. Calibrated aluminium attenuators reduced the incident flux at the sample (determined from air-absorption-corrected ion-chamber measurements) by factors of ∼11, 46, 377 and 847, giving nominal dose rates for thaumatin of ∼3.3, 0.78, 0.095, and 0.042 MGy s^−1^, respectively. These dose-rate estimates do not include the effects of finite photoelectron mean free paths (Supporting Information §S5; Finfrock *et al.*, 2013 ▸; Nave & Hill, 2005 ▸; Sanishvili *et al.*, 2011 ▸) or fluorescence. These effects are independent of dose rate and are small for the beam size used here.

### 2.4. Diffraction data collection   

X-ray diffraction data were collected using a PILATUS3 300K detector with a maximum frame rate of 500 Hz. The detector was mounted with its lower edge just above the direct beam, and with its face tilted (at ∼22°) so that the center of the detector was normal to the diffracted beam direction at this position. For data acquisition with the unattenuated beam, the full 500 Hz frame rate was used; for attenuated beams the exposure time per frame was increased by the attenuation factor so that frames acquired with different attenuation settings were comparably exposed. Counts per pixel per frame in the brightest diffraction peaks were typically <10 000, giving a dead-time-corrected error in the 1 ms exposure at the highest frame of less than 10% (Supporting Information §S9). At a given position on a crystal, diffraction patterns were acquired at the attenuation-dependent frame rate for a total exposure time that gave a significant reduction (by a factor of ∼3 at 260 and 300 K) in the Bragg diffraction of the crystal. Between five and 35 diffraction time series were acquired from each crystal at each of the five attenuation settings by stepping the crystal through the beam in 20 µm steps. The crystal orientation was fixed, ensuring that a fixed crystal volume was illuminated throughout each frame series.

### 2.5. Data processing   

The ∼50 000 individual diffraction frames were processed using *DISTL* (Zhang *et al.*, 2006 ▸; Sauter, 2010 ▸), and separately using *XDS* (Kabsch, 2010 ▸), to extract Bragg peak positions and intensities. The frames were all ‘stills’ (*i.e.* recorded with zero oscillation) and captured roughly 40% of the full diffraction pattern. At 260 and 300 K thaumatin crystal mosaicities are typically very small (<0.01°). Each frame thus had at most a few hundred well exposed peaks. Attempts to index the frames were often unsuccessful, and parameter choices that allowed successful indexing did not give sensible mosaicity values. To quantify radiation damage, an integrated intensity for each frame was calculated using *DISTL* by summing the integrated spot intensities in pixel ADC units above the local background. As a check on these results, a measure of integrated intensity for each frame was calculated by summing peak *I*/σ values in the SPOT output of *XDS*. These two integrated intensities were then plotted *versus* frame number (proportional to dose) to obtain a ‘dose curve’. Similar metrics of integrated intensity have been used in previous high-dose-rate studies of radiation damage (Owen *et al.*, 2012 ▸, 2014 ▸). To assist in interpreting the dose curves, the *DISTL*- and *XDS*-generated intensities of each individual diffraction peak *versus* frame number for all ∼1300 dose series were plotted and manually inspected. All data and figures presented here are based on analysis using *DISTL*, but qualitatively and quantitively similar results were obtained using *XDS* (Supporting Information §S14).

## 3. Results and discussion   

### 3.1. Integrated intensity *versus* dose: non-exponential decays and a ‘lag phase’   

**Figure 1:**
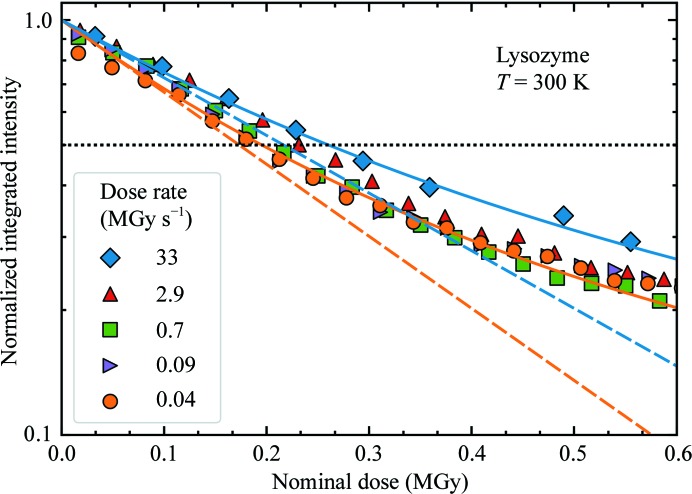
Representative semi-log plot of the integrated intensity in diffraction peaks *versus* dose at several dose rates, acquired from a single, fixed-orientation lysozyme crystal at 300 K; Supplementary Fig. S5 shows 260 K data. Solid lines are single-parameter fits at the highest and lowest dose rates based on the model described here; dashed lines indicate the initial exponential trend. The intersection of the horizontal dashed black line with each dose curve determines the half-dose *D*_1/2_. Doses and dose rates in all figures are averages within the area of the FWHM of the Gaussian beam.

Fig. 1 ▸ and Supplementary Fig. S5 show representative plots of integrated intensity *versus* dose for five different dose rates at 300 and 260 K. At all temperatures, the dose curves have a roughly exponential dependence on dose (dashed lines) until the integrated intensity has decreased to roughly half its initial value (at a dose called the half-dose, *D*_1/2_). However, at larger doses the curves deviate above the initial exponential trend. This deviation is observed at all dose rates for all crystals of lysozyme and thaumatin at all temperatures. Intensity *versus* dose curves with comparable shapes and initial slopes are obtained when integrating only the 10, 25, 50 and 100 brightest peaks in each frame set, indicating that deviations from exponential behavior are not a consequence of background subtraction (except perhaps at very large doses, where the integrated intensity becomes very small). Attempts to force exponential behavior by adjusting background subtraction give decays with dose that are a factor of ∼3 more rapid than in previous measurements.

**Figure 2:**
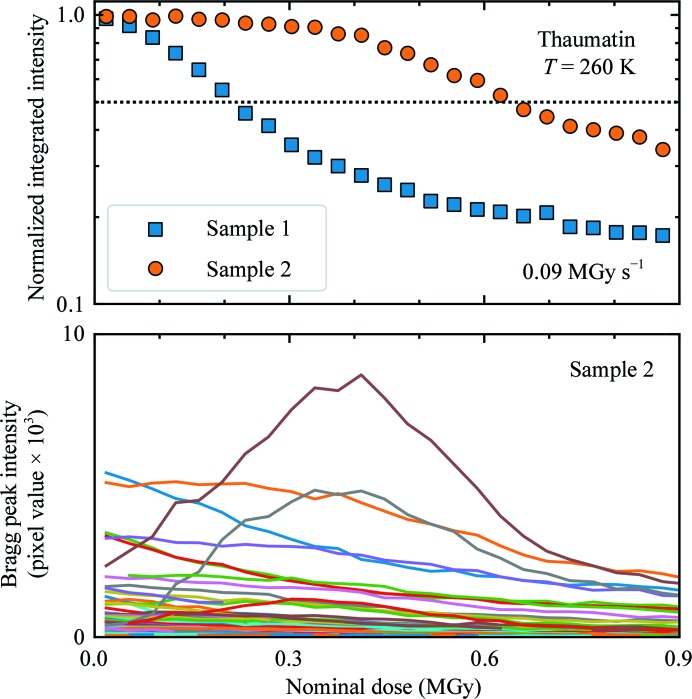
Top: representative integrated intensity *versus* dose data for two thaumatin crystals at 260 K for a dose rate of 0.09 MGy s^−1^. Each curve was recorded from one sample position. Sample 1 has an intensity variation with dose as in Fig. 1 ▸, while sample 2 has an initial plateau in intensity. Bottom: the intensity plateau for sample 2 results from initial growth with increasing dose of a subset of Bragg peaks that dominate the integrated intensity. Supplementary Fig. S6 shows similar data acquired at a dose rate of 36 MGy s^−1^.

Not all measured dose curves exhibit an initial exponential decay. As shown in Fig. 2 ▸, roughly 5% of 683 lysozyme dose curves and 25% of 627 thaumatin dose curves show an initial plateau or near-plateau. Similar behavior at dose rates above 1 MGy s^−1^ has been reported (Owen *et al.*, 2012 ▸, 2014 ▸) and the apparent delay in intensity decay has been described as a ‘lag phase’. These initial plateaus are observed here at all dose rates and temperatures. Their width *in time* varies according to the dose rate by a factor of ∼10^3^ at each temperature, but their width *in dose* is roughly consistent (Owen *et al.*, 2014 ▸), generally ∼1/4 to 1/2 the half-dose *D*_1/2_. Some crystals, each with a different orientation, yield far more dose curves with plateaus than others. Of the seven thaumatin crystals examined at 260 K, one shows plateaus or small initial slopes in all 84 of its dose curves, whereas another shows plateaus or small initial slopes in only five of its 51 dose curves.

### 3.2. Bragg peak intensities *versus* dose   

Plots of individual Bragg peak intensities *versus* dose aid in interpreting integrated intensity plots, allowing artefacts owing to sample motion and spurious diffraction peaks from, for example, salt to be identified. These plots also provide insight into the origin of the plateaus in Fig. 2 ▸ (top). As illustrated in Fig. 2 ▸ (bottom), in *every* case in which the integrated intensity *versus* dose exhibits an initial plateau or near-plateau, a few to several intense Bragg peaks have intensities that initially increase rapidly or show plateaus with dose. The eventual radiation-damage-induced decay of these peaks occurs at doses that roughly match the width of the integrated intensity plateau. When only the brightest ten or 25 peaks in each frame are integrated the integrated intensity can show a significant initial increase with dose before decaying at larger doses. Similarly, in every case where plateaus *versus* dose are not observed, at most one or two intense peaks show initial increases. These trends are not an artefact of background subtraction and integration, and are clearly visible in the original diffraction frames (Supplementary Fig. S7).

### 3.3. Intensity fluctuations at high dose and frame rates   

As shown in Supplementary Fig. S8, at 260 K and especially at 300 K, integrated intensity *versus* dose curves acquired at the highest dose rate and detector frame rate (500 Hz) consistently show fluctuations, where the integrated intensity increases for one or two frames before dropping back down to the overall trend, that are brief in both dose and time. At the second highest dose rate and frame rate (83 Hz) smaller jumps are observed, and at the lowest dose and frame rate (1.2 Hz) the dose curves are generally smooth. At all dose rates fluctuations are observed only at doses comparable to or beyond the half-dose *D*_1/2_, and the typical separation in dose between individual fluctuations in a given dose curve is of the same order as the half-dose. No such fluctuations are observed at any dose rate for either protein at 100 K, although these data do not extend beyond the (large) half-doses at this temperature.

### 3.4. Temperature- and dose-rate-dependent half-doses   

**Figure 3:**
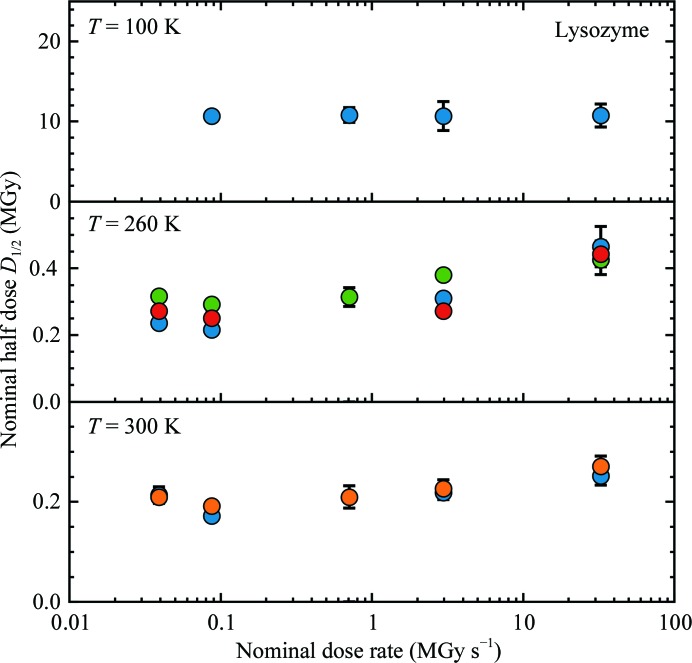
Half-dose *versus* dose rate for tetragonal lysozyme at 100, 260 and 300 K. Similar plots are obtained for thaumatin, and half-doses are summarized in Supplementary Table S1. Each dose-rate point represents an average of half-doses determined from between five and 35 dose curves obtained from different positions on each sample. The different symbols at each temperature indicate data from different samples. The error bar on each point represents the corresponding standard deviation.

Fig. 3 ▸ and Supplementary Table S1 give the measured nominal half-doses *D*_1/2_ *versus* nominal dose rate and temperature for lysozyme and thaumatin. Half-doses were obtained from an exponential fit to the initial decay of each dose curve, down to where the intensity decreased to roughly one-half of its initial value. Dose curves exhibiting initial plateaus were excluded from this analysis, although fits to these curves over an equivalent dose range that excluded the initial plateau yielded quantitatively similar half-doses to those in Fig. 3 ▸. At *T* = 100 K, the half-dose is independent of dose rate over the entire dose-rate range. At 260 and 300 K, a clear dose-rate dependence is observed for both proteins. Using the unattenuated beam with a nominal dose rate of 33–36 MGy s^−1^ increases the half-dose by factors of ∼1.5–2 at 260 and 300 K relative to data collected at dose rates of less than 100 kGy s^−1^. This increase in half-dose is real and is not a detector artefact caused by large incident photon fluxes per pixel (Supporting Information §S9). At a nominal dose rate of ∼36 MGy s^−1^, the half-dose is reached in ∼6 ms at 300 K and in ∼10 ms at 260 K. Consequently, some radiation damage can be outrun and the amount of data collected per crystal increased by collecting diffraction data on this time scale.

### 3.5. Form of the intensity *versus* dose curves   

The observed form of the intensity *versus* dose curves is a consequence of non-uniform irradiation (Supporting Information §S12) provided by Gaussian profile microbeams and the resulting ‘hole burning’. Consider the following model. Let *F*(**r**, *t*) be the incident flux density (in photons m^−2^ s^−1^) at crystal position **r** and time *t*, and *D*(**r**, *t*) be the dose (in J kg^−1^) delivered at **r** from *t* = 0 to *t*, which depends on the incident flux density, the X-ray energy and the crystal composition. Let *S*[*D*(**r**, *t*)] be the diffracted flux per unit illuminated crystal volume per unit incident flux density at position **r** and time *t* (averaged over all reflections, proportional to the integrated intensity). *S*[*D*(**r**, *t*)] depends on how the diffracting power of the crystal at position **r **is reduced by damage caused by the dose *D*(**r**, *t*). Assume an exponential decay, *S*[*D*(**r**, *t*)] ∝ exp[−*D*(**r**, *t*)/*D*_e_], where the local half-dose (corresponding to the value measured using a uniformly irradiated crystal) is *D*_1/2,local_ = *D*_e_ln(2). Further assume that the incident flux density *F*(**r**, *t*) is time-independent (the beam flux density is constant and the crystal is not rotated or translated) and cylindrically symmetric about the beam center, and that the crystal is thin so that X-ray attenuation along the beam path can be neglected. The total diffracted flux from the sample at time *t* is then 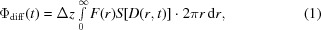
where Δ*z* is the crystal thickness along the beam and the dose *D*(*r*, *t*) = *ktF*(*r*), where *k* is a constant that depends on the X-ray energy and the sample composition.

**Figure 4:**
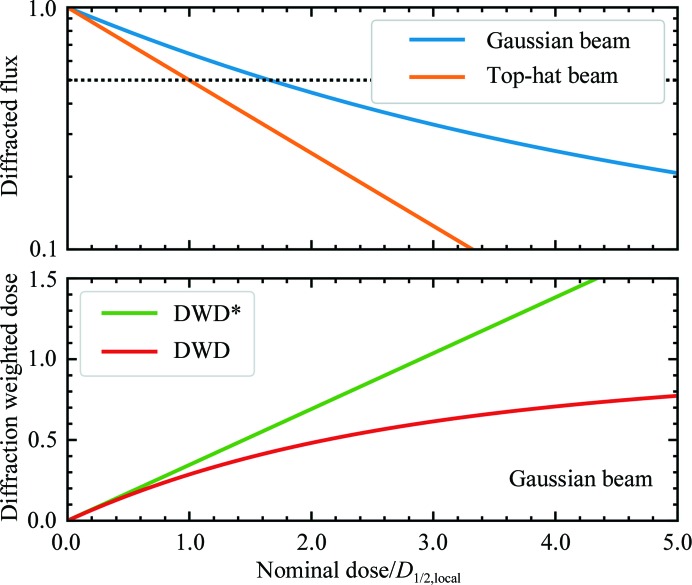
Top: diffracted flux from a crystal, proportional to the integrated intensity in Bragg peaks, *versus* dose calculated for a Gaussian beam and for a beam with a top-hat (rectangular) profile of width equal to the Gaussian FWHM, for a fixed crystal orientation during irradiation. The local decay of diffraction with dose is assumed to be exponential with a half-dose *D*_1/2,local_ equal to that measured with a top-hat profile beam. Bottom: diffraction-weighted dose DWD* (Zeldin, Brockhauser *et al.*, 2013 ▸) and DWD (as revised here) *versus* normalized dose for a Gaussian beam and a fixed crystal.

Fig. 4 ▸ (top) shows the resulting diffracted flux, which is proportional to the integrated intensity measured by the detector, *versus* normalized dose for a Gaussian profile X-ray beam, 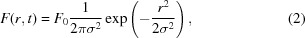
and a top-hat profile beam with the same FWHM and same total flux. For the Gaussian beam, the decay with dose is roughly exponential until the diffracted flux (integrated intensity) has decayed to roughly half its initial value, and is then much more gradual than this initial trend would predict. As shown in Fig. 1 and Supplementary Fig. S5, the calculated functional form provides a reasonable fit to integrated intensity *versus* nominal dose data for all dose rates at all temperatures studied here. Deviations of the fit above the data at large doses/small intensities are most likely to be owing to the decreased diffraction resolution and the increase in effective half-dose with decreasing resolution (Howells *et al.*, 2009 ▸), to deviations of the beam shape from a strict Gaussian, and possibly to errors in background subtraction. The calculated half-dose, using the average dose delivered in the FWHM of the beam, is *D*_1/2_ = 1.66*D*_1/2,local_, where *D*_1/2,local_ is the ‘true’, local half-dose.

**Figure 5:**
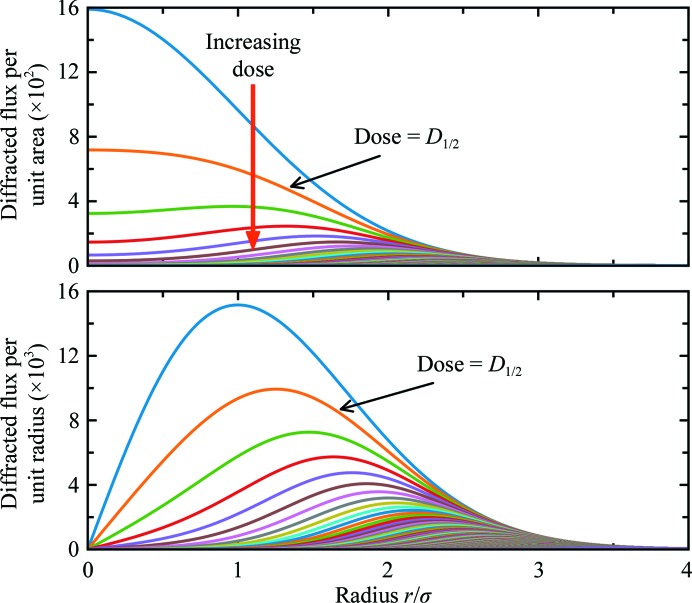
Calculated diffracted flux per unit beam area (top) and per unit beam radius *versus* radius (bottom) for equally spaced nominal doses for a Gaussian beam and a locally exponential decay of diffraction with dose.

Fig. 5 ▸ illustrates the origin of the non-exponential form of the calculated and observed dose curves. The incident flux density and dose vary with *r* within the illuminated crystal region. Initially, the diffracted flux per unit illuminated crystal area in Fig. 5 ▸ (top) is strongest at *r* = 0 and in the strongly illuminated core within the FWHM. However, since the core receives the largest dose rate, it is the most rapidly damaged, and diffraction from it fades most rapidly. Near the half-dose in Fig. 4 ▸, the diffracted flux per unit area in Fig. 5 ▸ has flattened near *r* = 0. At larger average doses radiation damage has burned a ‘hole’ near *r* = 0, and the most strongly diffracting regions are at larger radii. Fig. 5 ▸ (bottom) shows the (circumferentially integrated) diffracted flux per unit radius. The region near *r* = 0, despite producing the largest initial flux density, has a small area and contributes little to the total diffracted flux. The peak flux per unit radius moves from *r*/σ = 1 to larger *r* as irradiation proceeds, with its shift per unit dose (time) decreasing with increasing dose.

Non-exponential intensity decays with dose or illumination time have been observed in most previous radiation-damage studies of protein crystals (Blake *et al.*, 1962 ▸; Hendrickson, 1976 ▸; Sliz *et al.*, 2003 ▸; Warkentin & Thorne, 2010*a* ▸; Owen *et al.*, 2014 ▸; Liebschner *et al.*, 2015 ▸). When Bragg peak intensities within narrow resolution shells have been plotted *versus* dose, higher resolution shells deviated from exponential behavior at lower doses. These data have been analysed using models (Blake *et al.*, 1962 ▸; Hendrickson, 1976 ▸; Sygusch & Allaire, 1988 ▸) that consider local dose-dependent transitions between undamaged protein, partially disordered protein and fully amorphous protein, and that give rise to a locally non-exponential dose response. However, in all experiments the crystals were non-uniformly irradiated owing to a non-uniform beam profile and/or owing to sample rotations during illumination. With an underlying exponential intensity–dose relation, non-uniform illumination alone can explain the qualitative shape of the observed intensity *versus* dose curves (Supporting Information §S12). Resolution-dependence of the non-exponential behavior results because the effective local half-dose decreases with increasing resolution (Howells *et al.*, 2009 ▸). Consequently, the experimental form of the local dose response and its implications for damage mechanisms must be re-evaluated in light of the present analysis.

### 3.6. Quantifying radiation damage: half-doses and average dose state   

In routine crystallographic practice, the crystal is rotated instead of fixed, the X-ray beam is often smaller than the crystal and the crystal volume illuminated by the beam changes substantially with orientation (Zeldin, Gerstel *et al.*, 2013 ▸), so that different regions of the crystal receive different doses during data-set collection. Consequently, the photons recorded in a given frame will come from crystal regions that have received different doses, are in different damage states and that differ both in their resolution and in specific structural details owing to molecular damage.

A diffraction-weighted dose (denoted here by DWD*, for reasons that will become clear shortly) has been proposed as a better metric of the damage state in such cases of non-uniform irradiation (Zeldin, Brockhauser *et al.*, 2013 ▸). DWD* is defined as 
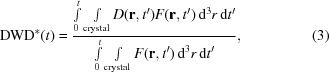
where *F*(**r**, *t*) is the incident flux density and *D*(**r**, *t*) is the total cumulative dose at *crystal* position **r** at time *t*. DWD* weights the dose delivered to each region of a crystal by the amount of X-ray illumination that it receives, which is assumed to be proportional to its contribution to the measured diffraction. Regions that are weakly or transiently illuminated receive a small dose, and the contribution of their dose to the DWD* is down-weighted. For a Gaussian profile X-ray beam and fixed crystal orientation as used here, *F*(**r**, *t*) = *F*(**r**) and *D*(**r**, *t*) = *D*(**r**), DWD* is 0.25 times the maximum dose *D*_max_ ∝ *F*(**r** = 0) at the beam center and 0.35 times the average dose delivered in the FWHM of the beam. The ratio of DWD* to *D*_max_ is constant, independent of the irradiation time. Consequently, plots of integrated diffraction intensity *versus* DWD*, *D*_max_ or average dose all have the same functional form.

However, DWD* is in fact an *incident flux density-weighted dose*: it assumes that the diffracted flux at time *t* depends only upon the incident flux and not on the accumulated dose. Consequently, it does not capture the dose state of the crystal regions that are responsible for diffraction at time *t*. For example, suppose a crystal is irradiated by a small square beam for a long time *t*_1_, receiving a large dose *D*_1_ such that the illuminated region ceases diffracting. If the beam is translated by half its width, DWD* will initially be *D*_1/2_, even though all of the diffraction comes from previously unirradiated and thus undamaged crystal.

A true diffraction-weighted dose (DWD) can be defined as
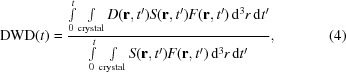
where the denominator gives the total diffracted intensity. The difference between DWD and DWD* is evident in Fig. 5 ▸ (bottom), which was calculated assuming a fixed-orientation sample illuminated using a Gaussian profile beam. DWD* increases linearly with dose. The true DWD has an initial roughly linear increase and then bends over, becoming nearly independent of dose at large doses. In other words, the dose that has been received by *those regions of the crystal that dominate diffraction at time t* becomes nearly independent of the average dose delivered to the crystal as a whole at large crystal doses. The reason for this surprising behavior is evident from Fig. 5 ▸ (bottom): as irradiation proceeds, the strongest diffraction comes from more weakly damaged regions at larger *r* values in the Gaussian profile, and there is much more sample volume per unit *r* at large *r* than at small *r*. When the diffracted intensity has decreased to half of its initial, zero-dose value, DWD* overestimates the true DWD by 35%, and this increases to 77% when the intensity is 30% of its initial value. For general incident X-ray beam profiles and sample-irradiation patterns, DWD provides a more relevant and lower estimate of the dose state of the sample, and is a key metric for optimizing data-collection protocols.

### 3.7. Origin of anomalous intensity variations and the ‘lag phase’   

Rather than indicating a delayed onset of radiation damage, initial plateaus in integrated intensity *versus* nominal dose, as in Fig. 2 ▸, result from damage-induced redistribution of electron density in reciprocal space. As a crystal is damaged, its unit-cell dimensions, the width of its unit-cell size distribution and its mosaicity all increase (Ravelli & McSweeney, 2000 ▸; Ravelli *et al.*, 2002 ▸; Garman & Owen, 2006 ▸; Shimizu *et al.*, 2007 ▸; Holton, 2009 ▸; Garman, 2010 ▸; Rajendran *et al.*, 2011 ▸; Coughlan *et al.*, 2017 ▸). In reciprocal space, these changes correspond to radial motion, radial broadening and axial broadening of reciprocal-lattice peaks about *q* = 0. Radiation-induced reciprocal-lattice peak broadening and motion can increase the overlap of some reciprocal-lattice peaks with the surface of the Ewald sphere, initially increasing the corresponding Bragg peak intensity, before the peak intensities decay owing to the overall decay of crystal diffracting power (described by the *B* factor); for peaks initially located on the surface of the Ewald sphere these effects cause more rapid intensity decay. The fraction of reciprocal-lattice peaks that are suitably positioned to generate initial Bragg peak brightening varies with crystal orientation, and in some orientations is sufficient to generate integrated intensity plateaus. The systematics of this Bragg peak brightening allow it to be distinguished from intensity changes owing to site-specific damage (Supporting Information §S10).

The generation of integrated intensity plateaus using this mechanism requires that the rate of mosaicity and/or cell-size distribution broadening with dose be ‘larger’ in some sense than the rate of overall loss of crystal diffracting power. Plateaus may be more prevalent for crystals with small initial mosaicities, illuminated by at most weakly diverging X-ray beams, held in fixed orientation during irradiation: the conditions that are most likely to prevail during serial synchrotron crystallography. Plateaus may be particularly acute when the beam is smaller than the crystal, as undamaged surrounding crystal regions then constrain irradiation-induced unit-cell expansion and may lead to fracturing and increased mosaic broadening. The underlying rapid and nonmonotonic evolution of individual Bragg peak intensities with dose (corresponding to a dose-dependent reflection partiality) will complicate the estimation of structure factors, especially when crystal diffraction is weak so that peak-intensity evolution with dose cannot be reliably recorded.

### 3.8. Origin of intensity fluctuations in high dose-rate and frame-rate data collection   

The salient features of the integrated intensity fluctuations with dose shown in Supplementary Figs. S8 and S9 are (i) they appear only after the sample has received a substantial dose, of the order of the nominal half-dose, (ii) they have the largest amplitude for data collected with the highest dose rate and shortest frame period, (iii) they always involve a transient increase in intensity, but do not rise to the zero-dose intensity, and (iv) most or all diffraction peaks in a given frame fluctuate in the same way. The fluctuations are not observed at low doses, regardless of dose rate, and are not obviously present in data collected at low dose and frame rates. (i) indicates that these fluctuations are associated with radiation damage, and that a minimum dose/amount of damage is required. (ii) suggests that the timescale for the fluctuations is short: on the order of milliseconds. (iii) and (iv) suggest that they arise from a small (∼200 nm or 0.03°) motion of the crystal that brings a small volume of less-exposed and less-damaged crystal into the beam.

Intensity fluctuations may arise from crystal and mounting loop ‘quakes’ in response to gas-bubble formation and bursting. Irradiation cleaves off H atoms (Leapman & Sun, 1995 ▸; Meents *et al.*, 2010 ▸) that, at sufficiently high temperature, can recombine to form H_2_. When crystals that have received a large dose at 100 K (where free radicals and hydrogen are immobile) are warmed to 300 K by blocking the cold gas stream, rapid radical diffusion, reaction and H_2_ production can lead to gas bubbling that destroys the crystal in a few seconds (Garman, 2010 ▸). When room-temperature crystals are irradiated at ∼35 MGy s^−1^, large doses are delivered in tens of milliseconds, the hydrogen concentration in the illuminated volume rises rapidly, and bubbles may nucleate, grow and burst, delivering impulses to the crystal and its mount that transiently bring less exposed crystal regions into the beam. Intensity fluctuations may also arise from crystal fracturing that relieves stress from damage-induced expansion of the irradiated volume. Both bubble growth/bursting and fracturing may generate significant uncertainties in structure-factor estimates based on microbeam data from weakly diffracting microcrystals.

### 3.9. Outrunning radiation damage using intense microbeams   

While the timescales for the chemical processes involved in radiation damage to protein crystals at and near room temperature are microseconds or shorter, the timescales for structural relaxations in response to chemical damage – including side-chain rotations, main-chain displacements and unfolding, molecular displacements and rotations within the unit cell, and the longer range lattice distortions responsible for the increase in mosaicity and unit-cell volume – may be much longer, especially for motions involving many atoms that may have a much larger effect on diffracted intensities than, for example, the breakage of bonds that precipitate the motions (Warkentin *et al.*, 2013 ▸, 2014 ▸). At room temperature, the rate of diffraction-spot fading with dose varies between proteins by a factor of ten or more, even though the extent of chemical damage per unit dose should be similar, suggesting the importance of structural relaxations (Warkentin *et al.*, 2014 ▸).

Several recent synchrotron-based experiments (Leiros *et al.*, 2006 ▸; Southworth-Davies *et al.*, 2007 ▸; Warkentin *et al.*, 2012 ▸; Owen *et al.*, 2012 ▸, 2014 ▸) have probed the dose-rate dependence of radiation damage and the feasibility of outrunning some fraction of damage by using intense microbeams and fast data collection. The results have been inconclusive. Reported increases in crystal lifetimes using large dose rates have varied substantially, and detector undercounting when photon fluxes per pixel are large has been suggested as a source of apparent crystal lifetime increases at the highest dose rates (Supporting Information §S9).

The present large and complete data set, which spans 26 crystals and more than 1300 intensity *versus* dose data sets, and the accompanying analysis establish that it is possible to outrun some damage at and near room temperature (but not at 100 K). At the largest nominal dose rate (∼35 MGy s^−1^), the half-dose at and near 300 K is larger by a factor of roughly 1.5–2 than at typical crystallographic dose rates of ∼10 kGy s^−1^ for both thaumatin and lysozyme. In serial crystallography, this increase in crystal lifetime should translate into a comparable reduction in the number of required crystals and thus in data-collection time per complete structural data set. Cooling to 260 K gives another factor of 1.5 increase in lifetime, and additional microfocusing to the 1 µm range (allowing a significant fraction of photoelectrons to carry their energy out of the illuminated volume) could give an additional factor of ∼2.

Together, the combination of intense microfocused synchrotron X-ray beams and modest reductions in data-collection temperature below 300 K could allow a factor of 4–5 increase in data-collection throughput. Even larger increases may be possible for crystals with larger room-temperature radiation-sensitivities (Warkentin *et al.*, 2014 ▸).

## 4. Conclusions   

The evolution of diffracted intensities from protein crystals during irradiation by intense Gaussian X-ray microbeams is complex. Non-exponential integrated intensity decays, which have long been interpreted in terms of sequential damage models, can arise from non-uniformity in crystal illumination, owing to a non-uniform beam profile and/or owing to sample rotation during illumination. Non-uniform illumination produces non-uniform damage, and for Gaussian beams, to a surprising evolution of the diffraction-weighted crystal damage state with illumination time. Radiation-damage-induced reciprocal-space peak broadening can lead to plateaus or initial increases of individual peak and integrated intensities with dose, mimicking the effect of a delayed onset of damage. These and other effects described here significantly complicate the extraction of reliable structure factors from measured intensities. This will be especially true when data are collected to near and beyond the half-dose of the overall integrated intensity, and also to beyond the (smaller) half-dose of the highest resolution shells, and when the dose per frame is a substantial fraction of the half-dose, so that averaging over the nonmonotonic evolution of individual peak intensities occurs and extrapolation to zero-dose intensity values is not possible. These conditions are likely to prevail in serial synchrotron crystallography using microfocused beams and microcrystals. Consequently, optimizing data-collection protocols and maximizing the accuracy of extracted structure factors and structural models will require advances in modeling the complex spatio-temporal effects of radiation damage under these conditions.

## 5. Related literature   

The following references are cited in the Supporting Information for this article: Finfrock *et al.* (2010 ▸), Kriminski *et al.*, 2003 ▸), Stern *et al.* (2009 ▸), Warkentin *et al.* (2011 ▸) and Wei *et al.* (2000 ▸).

## Supplementary Material

Extensive additional data and information relevant to interpreting the results.. DOI: 10.1107/S2052252517013495/lz5016sup1.pdf
