# Supplementary material for: Lifetimes and spatio-temporal response of protein crystals in intense X-ray microbeams
Source: IUCrJ. 2017 Oct 13;4(Pt 6):785–94. doi: 10.1107/S2052252517013495 (PMC5668864; doi:10.1107/S2052252517013495)
Supplement: Supplementary file 1 [file m-04-00785-sup1.pdf]

IUCrJ

Volume 4 (2017)

Supporting information for article:

Lifetimes and spatio-temporal response of protein crystals in intense X-ray microbeams

Matthew A. Warkentin, Hakan Atakisi, Jesse B. Hopkins, Donald Walko and Robert E. Thorne

S1. Crystallization method and conditions

Tetragonal thaumatin and tetragonal lysozyme crystals were grown in 24-well plates using the hanging-drop vapour diffusion method. Purified powders of thaumatin and lysozyme (3× recrystallized) were purchased from Sigma-Aldrich (St Louis, Missouri, USA). Thaumatin was dissolved to a concentration of 25 mg ml^{-1} in 100 m*M* potassium phosphate buffer at pH 6.8, and a well s

olution prepared by adding 1 *M* sodium potassium tartrate to the same buffer. Lysozyme was dissolved in 0.5 M sodium chloride and a well solution containing 1 M sodium chloride prepared. 10 µl drops obtained by mixing 5 µl each of protein and well solution were suspended over 500 µl of well solution.

S2. Crystal handling and data collection times

The time between crystal mounting in oil on a loop and data collection was typically 5 minutes, and the time required for collection of all data sets from each crystal was typically 15-30 minutes. The oil alone, without any capillary enclosure, was sufficient to prevent crystal dehydration during this period, as was verified by monitoring unit cell parameters and by collecting data with oil-coated crystals contained in polymer capillaries.

S3. Spatial spread of radiation damage

Diffraction data was collected from a series of positions on each sample, separated by 20 µm. As a test, a sample at 300 K was irradiated one position with a large dose (several times the half-dose), and then diffraction measurements were acquired using a low dose in 10 and 20 µm steps along perpendicular lines meeting at that dosed position. These measurements confirmed that damage from each irradiated position did not extend to adjacent positions. All effects reported – including intensity fluctuations with dose at the highest dose rates and integrated intensity plateaus – were equally likely to be observed in newly irradiated crystal regions as when previously irradiated spots were nearby. As discussed in S5 below, the broadening of the damage footprint due to photoelectron escape at the X-ray energy used is small compared with the beam size. Free radical mean free paths at room temperature in the high protein density environment of a crystal should be less than 1 µm. More likely causes of damage spreading are inhomogeneous stresses associated with the internal pressure increase caused by generation of defects and hydrogen, and plastic lattice failure and cracking at larger doses. However, the irradiated spot area of ~ 3 µm × 5 µm ~ 15 $µm^2$ is small compared with the 20 µm × 20 µm ~ 400 $µm^2$ allocated to each measurement, and so the amount of "spread" damage in any adjacent spot will be tiny compared with damage due to direct irradiation of that spot.

S4. Visual manifestations of X-ray beam – crystal interactions

Microbeam irradiation had two visual consequences. First, the irradiated positions of each crystal became visible as an array of cylinders (Fig. S3), due to small (~10^{-4}) fractional changes in refractive index associated with radiation-induced changes in unit cell volume and density. Second, at the highest dose rates optical fluorescence from the region being irradiated was clearly visible using the beamline telescope and camera (Fig. S4), especially at T=100 K where the decay time of the fluorescence following irradiation was several seconds. The power (intensity) associated with this fluorescence was a small fraction of the ~2 mW (1.2×10^8 W/m^2) of the incident X-rays.

S5. Effect of photoelectron escape on dose estimates

For sufficiently small microfocused volumes (or sufficiently small crystals), X-ray generated photoelectrons and fluorescent photons may leave the illuminated volume and deposit their energy outside of it, reducing the dose received, with the photoelectrons carrying most of the energy. Measurements using 18.5 keV microfocused Gaussian profile beams found that for 2.7 μm FWHM and 5.35 μm beams, the integrated intensity loss per unit dose was ~0.65 and 0.8, respectively, of that obtained using a 15.6 μm beam . For the 10 keV X-rays used here, photoelectron ranges should be smaller by a factor of roughly 3 (Stern *et al.*, 2009; Finfrock *et al.*, 2013, 2010). The reduction in actual dose due to photoelectron escape within our 2.4 × 5.1 FWHM beams should be less than 10%. Note that this effect is independent of dose rate and only weakly temperature dependent, and so will not affect radiation sensitivity ratios at different dose rates and temperatures.

S6. Effect of diffraction peak integration parameter choices on half-dose estimates

Diffraction peak intensities depend on parameters used to model and integrate the peak and background. These choices also affect how individual and frame-integrated peak intensities vary with dose, and the calculated half-dose values at which the integrated diffraction intensity decreases to half its initial value. However, for comparably exposed frames – acquired at different dose rates, from different samples, or at different temperatures – the ratio of half-doses is relatively insensitive to these choices. As a check on effects of frame integration parameters, integrated intensities versus dose were calculated by integrating only the 10, 25, and 50 brightest peaks in each frame, and compared with results for integration of all peaks. Except for very weakly exposed frames acquired, e.g., at the edge of a crystal, or when a few very bright peaks behaved differently (e.g., showed a large initial intensity rise) than most other peaks, the intensity versus dose curves and half dose values from these different integrations were consistent.

S7. Effect of Gaussian beam anisotropy

Although the model presented here assumes a circularly symmetric 2D Gaussian beam, the quantitative predictions are the same for anisotropic beams of the form

$$F(r,t) = F_0 \frac{1}{2\pi\sigma_x\sigma_y} e^{-\left(\frac{x^2}{2\sigma_x{}^2}+\frac{y^2}{2\sigma_y{}^2}\right)},$$

and so apply to the reported data collected with a 2.4 × 5.1 μm beam.

S8. Previous experiments on the time and dose rate dependence of radiation damage

The dose rate and thus time dependence of damage, relevant to high flux density microcrystallography, has been controversial. Initial experiments at dose rates up to 10 kGy/s in most cases found no dose rate dependence. Observations of dark progression — an increase in damage while the X-ray beam is turned off — versus temperature between 180 K and 240 K revealed a temperature-activated component of damage whose time scale extrapolated to ~1 s at 300 K, suggesting that X-ray data collection on shorter timescales could allow some fraction of radiation damage to be outrun (Warkentin *et al.*, 2011). Experiments using ~50 μm beams found that dose rates of ~680 kGy/s gave damage-limited crystal lifetimes for thaumatin crystals at 260 K ~50% larger than at typical crystallographic dose rates of ~10 kGy/s (Warkentin *et al.*, 2012), and that dose rates approaching ~1 MGy/s increased 300 K lifetimes of three crystal systems by 30-80% (Owen *et al.*, 2012).

Using 10 and 20 μm microfocused beams and the same Pilatus3-300 K detector (i.e, the identical unit, not just the same model, loaned by Dectris) used in the present experiments, integrated intensity versus dose data was observed to exhibit an initial plateau or region of reduced slope, which was described as a "lag phase," for dose rates above 1 MGy (Owen *et al.*, 2014). Measuring crystal lifetime using D_{85}, the dose at which the integrated diffraction intensity is reduced to 85% of its initial value, thaumatin crystal lifetimes at 300 K measured using a dose rate of 1.32 MGy/s were found to be almost a factor of four larger than with a dose rate of 0.36 MGy/s. Combining data for bovine enterovirus serotype 2 (BEV2) from two experiments using different detectors, D_{85} at 5 MGy/s was found to be roughly 7 times larger than at dose rates below 500 kGy/s. These large increases in apparent crystal lifetime were due to initial intensity plateaus (the "lag phase"), and because initial intensity plateaus have a larger effect on D_{85} than on $D_{1/2}$. By examining individual diffraction peak intensities versus dose, the plateaus and "lag phase" are shown here to be an artefact of how diffraction data was collected. The plateaus and D_{85} values calculated when they are present do not indicate an initially reduced rate of radiation damage.

S9. Effects of detector saturation on measured intensities

At high incident (on the detector area) photon flux densities, single photon counting detectors undercount due to incoming pulse pile-up. Each pixel in the array has a finite dead time or retrigger time following detection of a photon before it can detect another photon, and this leads to increased undercounting as the incident flux increases. Provided that scattering from the sample itself does not change during each frame, the statistics of photon arrival, including effects of synchrotron bunch structure, can be modeled and a dead time correction applied to the "raw" detected counts to extend the detector's effective linear response range to higher count rates. The Pilatus3-300 K detector used here has a maximum usable incoming photon count rate per pixel in excess of 10 Mcps, with a dead-time corrected error at 10 Mcps of less than 10%.

The maximum measured count rate per pixel observed in the ~50,000 frames of the present experiments, obtained when using the unattenuated beam, was ~ 11 Mcps. To investigate possible errors introduced at large count rates, every pixel with count rates larger than 5 Mcps was flagged, and a histogram of these high count rate pixels generated for the first frame of every dose series (in which pixel count rates were usually largest). These histograms then allowed the integrated intensity and individual peak intensities versus dose curves to be compared based on first frame pixel count rates.

No effects whatsoever of maximum pixel count rates and number of high count rate pixels on the integrated or individual peak intensity versus dose curves were found. Between 16 and 50 dose curves were collected using the unattenuated beam from each crystal, each with the same crystal orientation but at a different position on the crystal. Due to variations in crystal thickness with position, some positions yielded first frames with up to 14 pixels having count rates >5 Mcps, while other positions had no high count rate pixels. These crystal positions all yielded quantitatively similar dose curves, half-doses, and individual peak intensity vs frame curves. There was also no correlation between maximum count rates per frame and the presence of plateaus in the dose curves. 39 of the 212 dose curves acquired from thaumatin crystals at the maximum dose and count rates showed initial plateaus or significantly reduced slopes, and none of the 271 dose curves acquired from lysozyme at the maximum dose rate showed plateaus. Plateaus were observed for all dose rates (attenuator settings), and at the lowest dose rate pixel count rates were ~10 kcps or smaller.

Pulse pile-up / dead time corrections can fail if the statistics of photon arrival during an exposure does not match model assumptions. This can happen during oscillations using very small mosaicity crystals, where a very bright peak may "flash" on and off in a time short compared with the pixel exposure time. Since the dead time correction is applied to the total recorded counts, it may under-correct counts recorded during the flash. In a dose series where the intensities of peaks decay from

frame to frame, undercounting due to such count correction errors will be largest in the initial, brightest frames, and so may cause an initial flattening of the dose curve.

Even though all the data collected here was from crystals in fixed orientations, sample position and orientation changes could in principle have occurred due to vibration in the gas stream (at 100 K and 260 K), and due to crystal sedimentation (at 260 K and especially 300 K). At 100 K, crystal mosaicities are large (~0.3° or more) and so count rates should be relatively immune to motions. At all temperatures, count rate corrections and undercounting could only have been an issue when using the unattenuated beam, for which the detector measurement time per frame was 1 ms. Only very large and/or high frequency motions could produce mosaic-width-size orientation changes on this timescale. To check for sample motions, intensity versus frame number plots for the diffraction peaks in all 1300 dose series were manually inspected. Since detector frame rates varied from 500 Hz to 1.2 Hz, these data were sensitive to motions on a wide range of time scales. In a few dose series, the amplitudes of individual peaks were observed to fluctuate with frame number, suggesting sample motion, but this was the rare exception, and these frames were excluded from our analysis.

S10. Origin of integrated intensity plateaus and site-specific radiation damage

Variations in relative diffraction peak intensities with dose, the cause of plateaus in integrated intensity observed here, can also arise from site-specific damage, i.e., from radiation-induced atomic displacements that are correlated between unit cells (e.g., breaking of disulphide bonds, reduction of metal centers) that result in changes in the underlying structure factors (Wei *et al.*, 2000). For fixed crystal orientation and fixed dose rate, bond breaking and other site-specific damage and thus the evolution of peak intensities with dose should be independent of crystal position, since the microscopic details of molecular damage should depend only on dose and crystal composition.

In fact, the rise and fall of individual Bragg peak intensities observed here is strongly position dependent, presumably because the evolution of mosaicity and/or lattice strain with dose at each position depends on local crystal thickness, proximity to crystal facets or edges, and the details of irradiation-induced fracturing and plastic failure within each illuminated volume.

The integrated intensity plateaus observed here and in Ref. (Owen *et al.*, 2014) were recorded using a Pilatus3-300 K detector, which was positioned with its lower edge just above the beam and recorded roughly 1/3 of the full diffraction pattern. Recording full frames (using, e.g., a Pilatus 6M detector) should modestly reduce but not eliminate the plateaus and the orientation-dependent variations in integrated intensity versus dose.

S11. Estimates of X-ray beam heating

In the present experiments, X-ray beam microfocusing increased the flux density and dose rate by a factor of ~10^3 relative to the X-ray beam exiting the monochromator, a gave a peak X-ray intensity at the sample of ~0.2 mW/μm^2. We previously showed that, provided the beam

is small compared with the crystal size, microfocusing produces only modest increases in temperature within the illuminated volume. For a cylindrical X-ray beam and cylindrical sample, the maximum steady-state temperature rise in the illuminated volume is (Warkentin *et al.*, 2012)

$$\Delta T(r_1) = \frac{D'\rho r_1^2}{2k}\left[\log\left(\frac{r_2}{r_1}\right) + \frac{k}{hr_2}\right]$$

Here D' is the dose rate, ρ is the sample density, k is the sample thermal conductivity, h is the heat transfer coefficient at the sample surface, r_1 is the beam radius, and r_2 is the sample radius. Using D' = 30 MGy/s, ρ = 1200 kg/m^3, k = 0.6 W/mK (the value for water at 300 K), h=290 W/m^2/K at 300 K (Kriminski *et al.*, 2003), r_1 = 2 μm, and r_2 = 50 μm gives $\Delta T \approx 6$ K. This is small compared to the temperature change required to appreciably affect the rate of radiation damage at all temperatures studied.

Note also that heating raises the crystal temperature, and that radiation damage per unit dose increases with temperature. Consequently, X-ray beam heating at the highest dose rates used here should make crystals appear more radiation sensitive; in fact, they are found to be less radiation sensitive than at lower dose rates.

Sample heating will be much larger if the beam and crystal sizes are comparable. For an upper bound estimate, assume a crystal size equal to the FWHM and that heating is adiabatic. With a dose rate of 30 MGy/s within the FWHM, this gives an initial heating rate of ~ 7 K/ms and a temperature rise during irradiation to the half-dose at T=300 K of ~100 K. For a beam size equal to the sample size (r_1=r_2), the above equation for a long cylindrical sample gives a steady state temperature rise of ~125 K; for a sample of length (along the beam) comparable to its diameter, heat transfer will be more effective than in the long-cylinder approximation leading to the above expression and the steady state temperature rise will be smaller.

S12. Origin of non-exponential decays of integrated intensities

Non-exponential decays of integrated intensities with dose or irradiation time, with more gradual decays observed at large doses/irradiation times, have been frequently observed in previous experiments using protein crystals, including those using X-ray microbeams. The non-exponential behavior has been analysed using models that consider local dose-dependent transitions between undamaged protein, partially disordered protein, and fully amorphous protein, which give rise to a locally non-exponential variation of diffracted intensity with dose. However, Gaussian X-ray beams – and, more generally, any spatially non-uniform X-ray dosing of the crystal – will give nonexponential decays of the diffracted intensity even if the underlying local relationship between diffracted intensity and dose is purely exponential. This is true even if the X-ray beam has a “top-hat” profile, and nonuniform irradiation is due to, e.g, crystal rotation or displacement.

For example, suppose a crystal is illuminated with a square, uniform profile beam and that the crystal is translated in a series of steps that are small compared with the beam size during irradiation. On first turning on the beam the intensity will decay exponentially with dose delivered to the crystal. But as the crystal is translated, the unexposed region that moves into the beam in each step will contribute more to the total diffracted intensity than previously exposed and damaged regions that remain in the beam. For doses per step interval that are comparable to the local half-dose, after a few steps essentially all the diffraction will come from newly illuminated crystal, the diffracted intensity (averaged over the time for one step) will become independent of dose delivered to the crystal and time, and the diffraction weighted dose DWD will become independent of dose and time. As a second example, suppose that a large crystal is illuminated by a smaller top-hat beam, that the crystal is repeatedly rotated through the same small (say, 5°) angular wedge during data collection, and that the integrated diffraction intensity collected during each rotation plotted versus time or total dose. As the dose in the central, continuously illuminated region of the wedge increases through the half-dose, more and more of the diffracted intensity from each wedge will come from the relatively undamaged crystal regions that are only transiently illuminated at the extreme limits of the rotation. The integrated intensity will then deviate upward from the exponential decay describing the local damage response. This wedge data collection mode was used in previous radiation damage studies by some of the present authors, where non-exponential decays were analysed using the local damage models.

Additional deviation from exponential decays at large doses will arise because there is no single half dose $D_{1/2,\mathrm{local}}$ or exponential decay constant D_e value that can describe the decay of Bragg peak intensities at all angles and resolutions. Low resolution Bragg peaks correspond to long wavelength Fourier components of the electron density, and much more dose and damage are needed to disrupt crystal structure and electron density on large length scales than on short length scales. This is discussed and experimentally demonstrated over three orders of magnitude in resolution by Howells et al. (Howells *et al.*, 2009). Note that this behavior is in some sense fundamental to radiation damage, and does not require invocation of a three-state model or any other particular model for the microscopic nature of damage.

S13. Implications for structure factor determination in the presence of radiation damage

As will be discussed in more detail elsewhere, the present results are of particular importance when data is collected to “large” doses, and/or when data collection involves substantial doses per frame. “Large” means comparable to the half-dose, which depends on the initial diffraction resolution of the crystal; crystals that initially diffract to high resolution have smaller half-doses and so are more likely to receive “large” doses. For data collection to large doses (e.g., during a rotation series), the complex spatiotemporal evolution of diffraction within the illuminated volume and the distribution of damage states within that volume will make it difficult to extract true structure factors from the measured Bragg peak intensities without modelling. In serial crystallography using high flux microfocused beams and

microcrystals, crystals may receive large doses in a single recorded frame, and the measured Bragg peak intensities will average over the complex spatio-temporal evolution of diffraction during that frame. Note also that models currently used to extrapolate measured structure factors to their zero-dose values do not account for the effects described here, and may lead to large errors when, e.g., the exposure per frame is not small.

S14. Comparison of diffraction intensities and half doses generated using DISTL and XDS

Both XDS and DISTL were used to identify and integrate individual diffraction peaks in our diffraction frames, which consisted of time/dose series with the crystal held in a fixed orientation. The intensities of those peaks were then summed to generate integrated intensity (across the frame) versus dose plots. With our parameter optimization efforts, XDS provided more accurate and consistent peak identification in our still frames. DISTL was more likely to identify spurious peaks, and was more likely to fail in processing individual frames in a dose series. However, background-subtracted peak intensities reported by XDS are I/σ values (W. Kabsch, private communication), not absolute intensities as in DISTL, and so integrating the XDS output for each frame added a background and thus resolution-dependent weighting to the intensities.

Despite this weighting of individual peak intensities, the XDS-derived integrated frame intensities and their dose and dose rate dependence had all the same qualitative features as were observed using DISTL results, including initial plateaus in integrated intensities and larger half-doses at the highest dose rates. Half doses calculated using XDS agreed to within 15% of those determined using DISTL. Figure S10 shows XDS results for integrated intensity versus dose for lysozyme at T=260 K, corresponding to the DISTL results shown in Fig. S5. Figure S11 shows XDS results for integrated intensity and individual peak intensities corresponding to the results in Fig. S6.

SI References

Finfrock, Y. Z., Stern, E. A., Alkire, R. W., Kas, J. J., Evans-Lutterodt, K., Stein, A., Duke, N., Lazarski, K. & Joachimiak, A. (2013). *Acta Cryst. D*. **69**, 1463–1469.

Finfrock, Y. Z., Stern, E. A., Yacoby, Y., Alkire, R. W., Evans-Lutterodt, K., Stein, A., Isakovic, A. F., Kas, J. J. & Joachimiak, A. (2010). *Acta Crystallogr. Sect. D*. **66**, 1287–1294.

Howells, M. R., Beetz, T., Chapman, H. N., Cui, C., Holton, J. M., Jacobsen, C. J., Kirz, J., Lima, E., Marchesini, S., Miao, H., Sayre, D., Shapiro, D. A., Spence, J. C. H. & Starodub, D. (2009). *J. Electron Spec. Rel. Phen.* **170**, 4–12.

Kriminski, S., Kazmierczak, M. & Thorne, R. E. (2003). *Acta Cryst. D*. **59**, 697–708.

Owen, R. L., Axford, D., Nettleship, J. E., Owens, R. J., Robinson, J. I., Morgan, A. W., Doré, A. S., Lebon, G., Tate, C. G., Fry, E. E., Ren, J., Stuart, D. I. & Evans, G. (2012). *Acta Cryst. D*. **68**, 810–818.

Owen, R. L., Paterson, N., Axford, D., Aishima, J., Schulze-Briese, C., Ren, J., Fry, E. E., Stuart, D. I. & Evans, G. (2014). *Acta Cryst. D*. **70**, 1248–1256.

Sanishvili, R., Yoder, D. W., Pothineni, S. B., Rosenbaum, G., Xu, S., Vogt, S., Stepanov, S., Makarov, O. A., Corcoran, S., Benn, R., Nagarajan, V., Smith, J. L. & Fischetti, R. F. (2011). *Proc. Natl. Acad. Sci. U. S. A.* **108**, 6127–6132.

Stern, E. a, Yacoby, Y., Seidler, G. T., Nagle, K. P., Prange, M. P., Sorini, A. P., Rehr, J. J. & Joachimiak, A. (2009). *Acta Crystallogr. D. Biol. Crystallogr.* **65**, 366–374.

Warkentin, M., Badeau, R., Hopkins, J. B., Mulichak, A. M., Keefe, L. J. & Thorne, R. E. (2012). *Acta Crystallogr. Sect. D Biol. Crystallogr.* **68**, 124–133.

Warkentin, M., Badeau, R., Hopkins, J. & Thorne, R. E. (2011). *Acta Crystallogr. Sect. D Biol. Crystallogr.* **67**, 792–803.

Wei, K, M., Ravelli, R. B. G., Kryger, G., McSweeney, S., Raves, M. L., Harel, M., Gros, P., Silman, I., Kroon, J. & Sussman, J. L. (2000). *PNAS*. **97**, 623–628.

Table S1 Half doses versus dose rate at 100 K, 260 K, and 300 K for tetragonal lysozyme and thaumatin crystals. Each half dose value is an average of values obtained from between 5 and 35 positions on each crystal and, at 260 and 300 K, multiple crystals. The uncertainties represent the standard deviations of all measured values at each temperature.

	**Dose Rate (MGy/s)**	**Half Dose $D_{1/2}$ (MGy) T=100 K**	**Half Dose $D_{1/2}$ (MGy) T=260 K**	**Half Dose $D_{1/2}$ (MGy) T=300 K**
**lysozyme**	0.039	-	0.27 ± 0.01	0.21 ± 0.02
	0.087	10.7 ± 0.5	0.25 ± 0.02	0.18 ± 0.01
	0.71	10.8 ± 0.9	-	0.21 ± 0.02
	2.97	10.7 ± 1.8	0.32 ± 0.02	0.22 ± 0.02
	32.6	10.8 ± 1.4	0.44 ± 0.08	0.26 ± 0.03
**thaumatin**	0.042	13.8 ± 0.5	0.41 ± 0.02	-
	0.095	12.6 ± 0.2	0.39 ± 0.04	0.16 ± 0.04
	0.77	-	-	0.20 ± 0.05
	3.24	14.5 ± 0.6	0.43 ± 0.04	0.22 ± 0.02
	35.6	12.3 ± 0.9	0.49 ± 0.05	0.35 ± 0.01

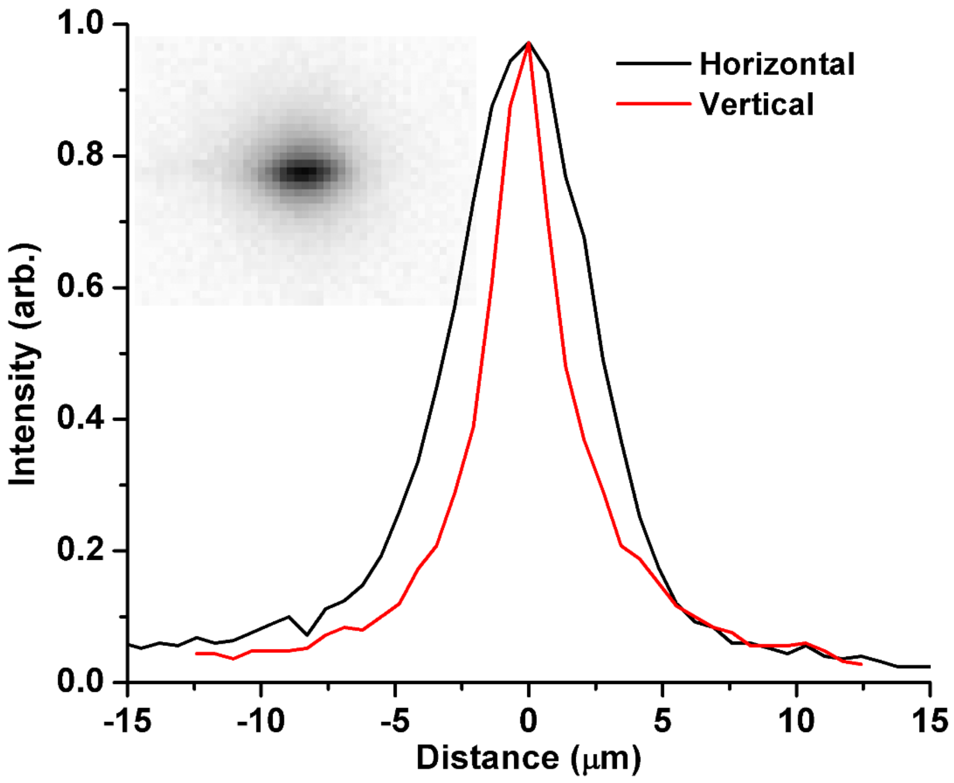

Figure S1 Microfocused X-ray beam profile determined by scanning a GaAs wafer edge through the beam and recording the detected intensity. The inset shows an image of the attenuated beam.

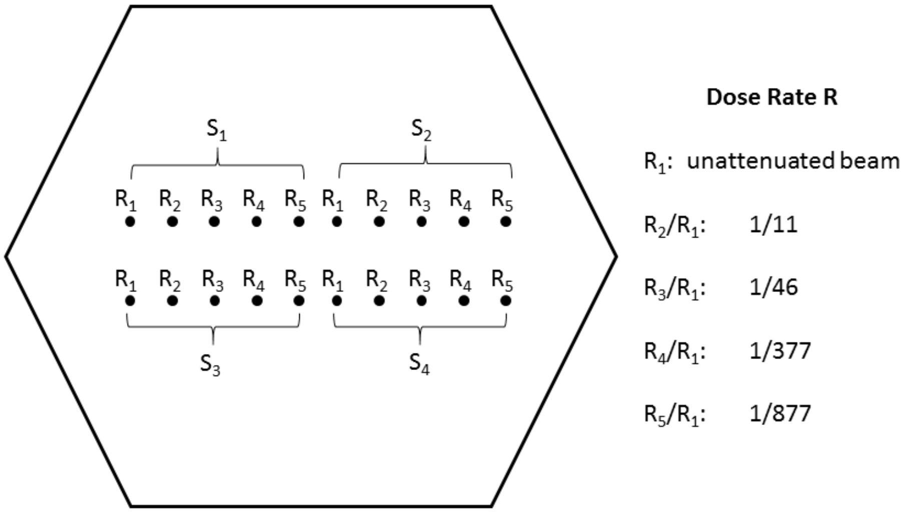

Figure S2 Example crystal irradiation pattern. Solid black dots represent irradiated positions. Each dose rate series S involved irradiating the crystal at 5 consecutive positions (separated by 20 μm) with each of the five dose rates R, obtained by attenuating the microfocused beam.

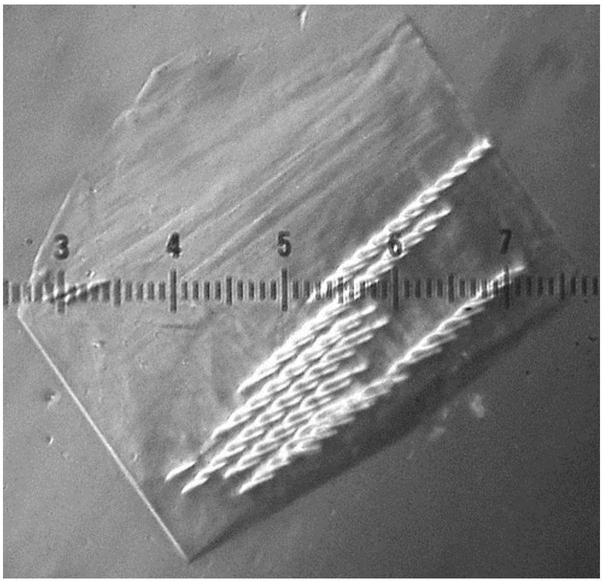

Figure S3 A thaumatin crystal, removed from the cryoloop, after microbeam irradiation following the general scheme illustrated in Figure S2. The major scale divisions are approximately 100 μm. The crystal was translated by 20 μm between measurement of each intensity versus dose frame set. Each position was irradiated using a different dose rate, but total exposure times varied such that all positions received the same dose, consistent with their similar appearances.

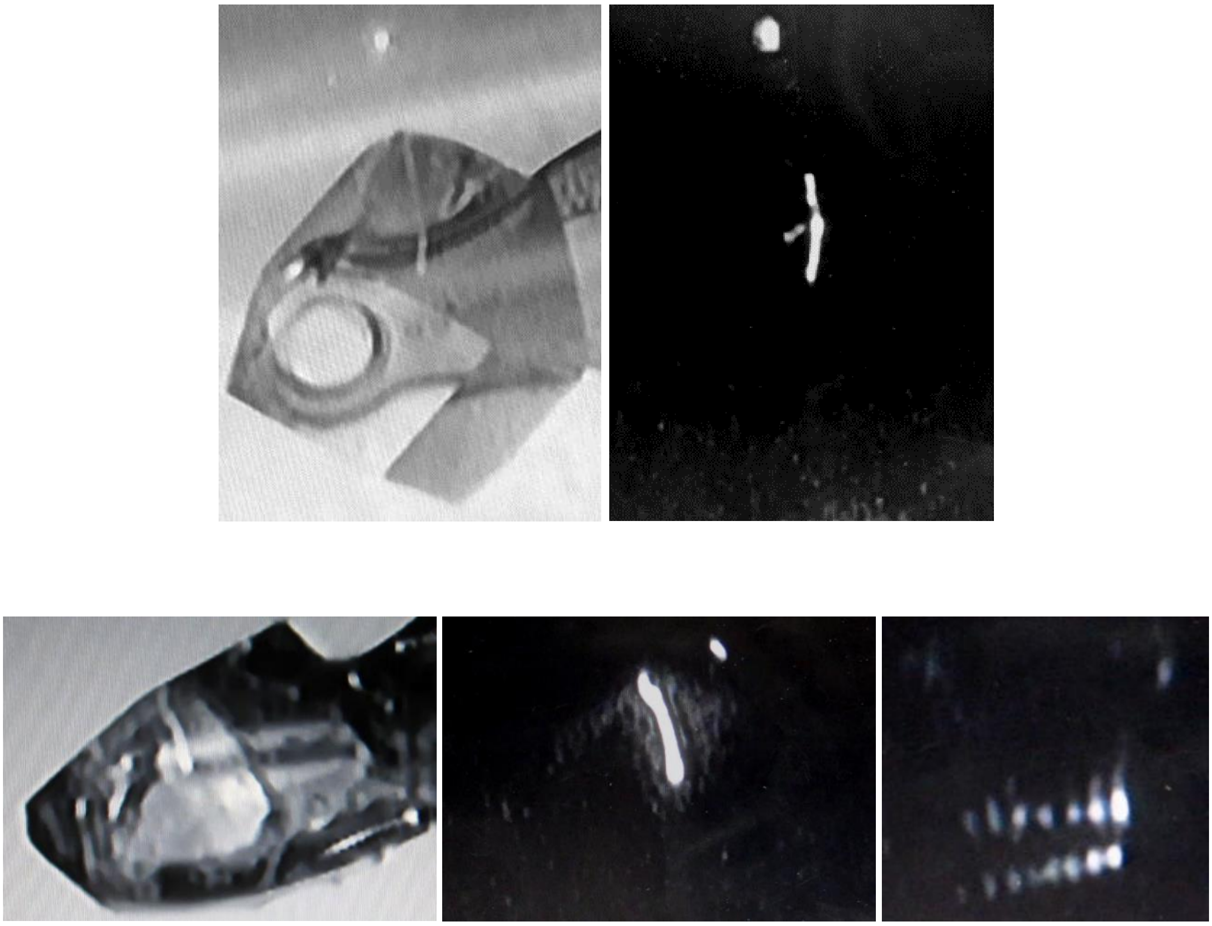

Figure S4 The flux density in the unattenuated beam generated optically visible fluorescence in the X-ray illuminated region at all temperatures. (Top) A lysozyme crystal at 260 K, with the beam hutch lights on (left) and off (right), as viewed through the beamline telescope. (Bottom) A lysozyme crystal at 100 K. The right-hand frame shows the fluorescence afterglow following six consecutive exposures separated by 1 s. At 260 K the fluorescence faded immediately, but at 100 K it persisted for ~6-8 seconds. Shape and brightness variations of the cylindrical illuminated crystal regions are due to reflection and refraction from crystal facets.

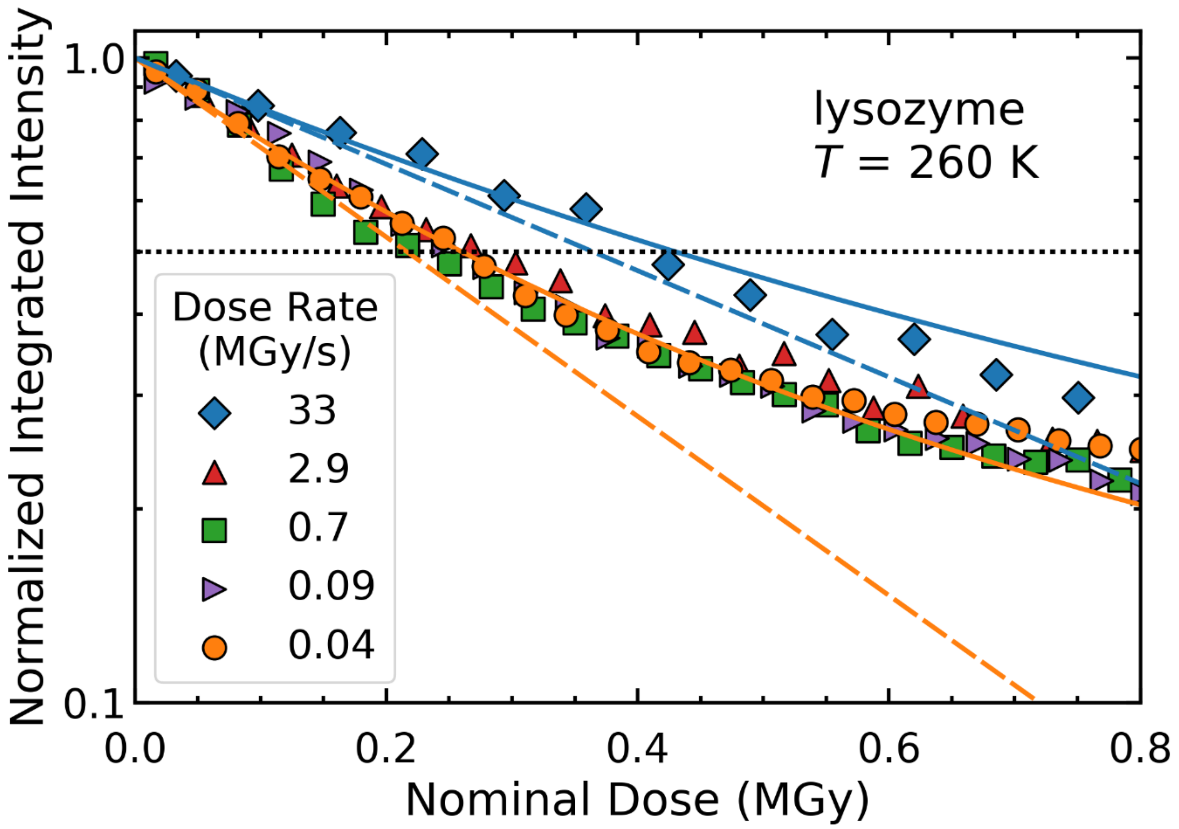

Figure S5 Representative semi-log plot of the integrated intensity in diffraction peaks versus dose at several dose rates, acquired from a single lysozyme crystal at 260 K; data at 300 K are shown in Fig. 1. The crystal was held in a fixed orientation and data for different dose rates was collected from five different positions. The intensity has an initial exponential decay (dashed lines), but varies much more gradually with dose at larger doses. At large doses/long times the data for different dose rates must converge. The solid lines are single-parameter fits to data at the highest and lowest dose rates based on the model described here. The intersection of the dotted black line with each dose curve determines the corresponding half-dose $D_{1/2}$. At 260 K the half-doses at all dose rates are larger than at 300 K. Doses and dose rates in all figures are averages within the area of the Gaussian beam's FWHM.

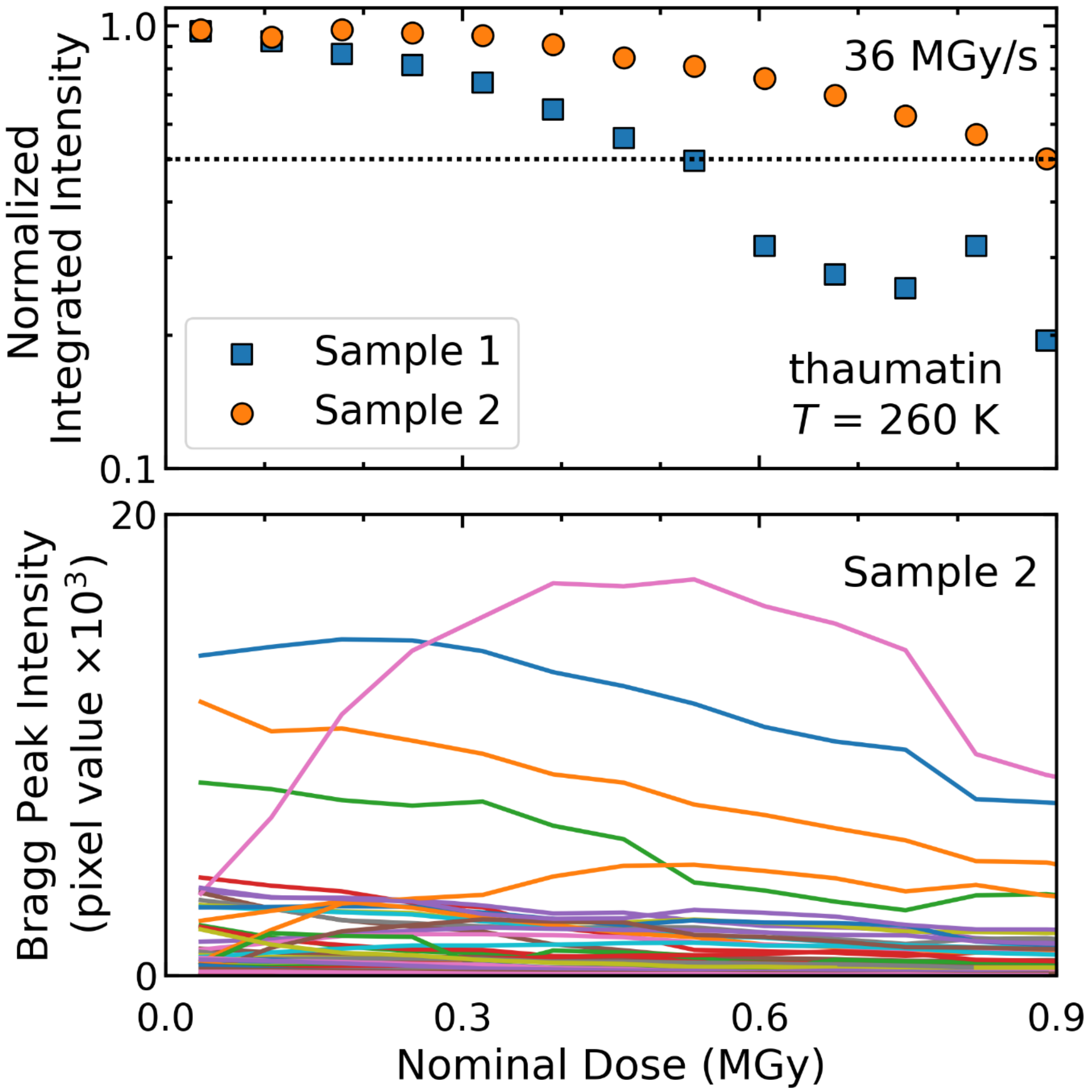

Figure S6 (Top) Representative intensity vs dose curves for two thaumatin crystals at 260 K for a nominal dose rate of 36 MGy/s. Each curve was recorded from one sample position. Sample 3 shows an intensity variation with dose as in Fig. 1, while Sample 4 exhibits an initial plateau in intensity. (Bottom) For samples and positions that yield plateaus or near plateaus in integrated intensity, a few to several diffraction peaks show a strong initial increase in intensity with dose, before decaying at larger doses. Similar data acquired at a dose rate of 0.09 MGy/s are shown in Fig. 2.

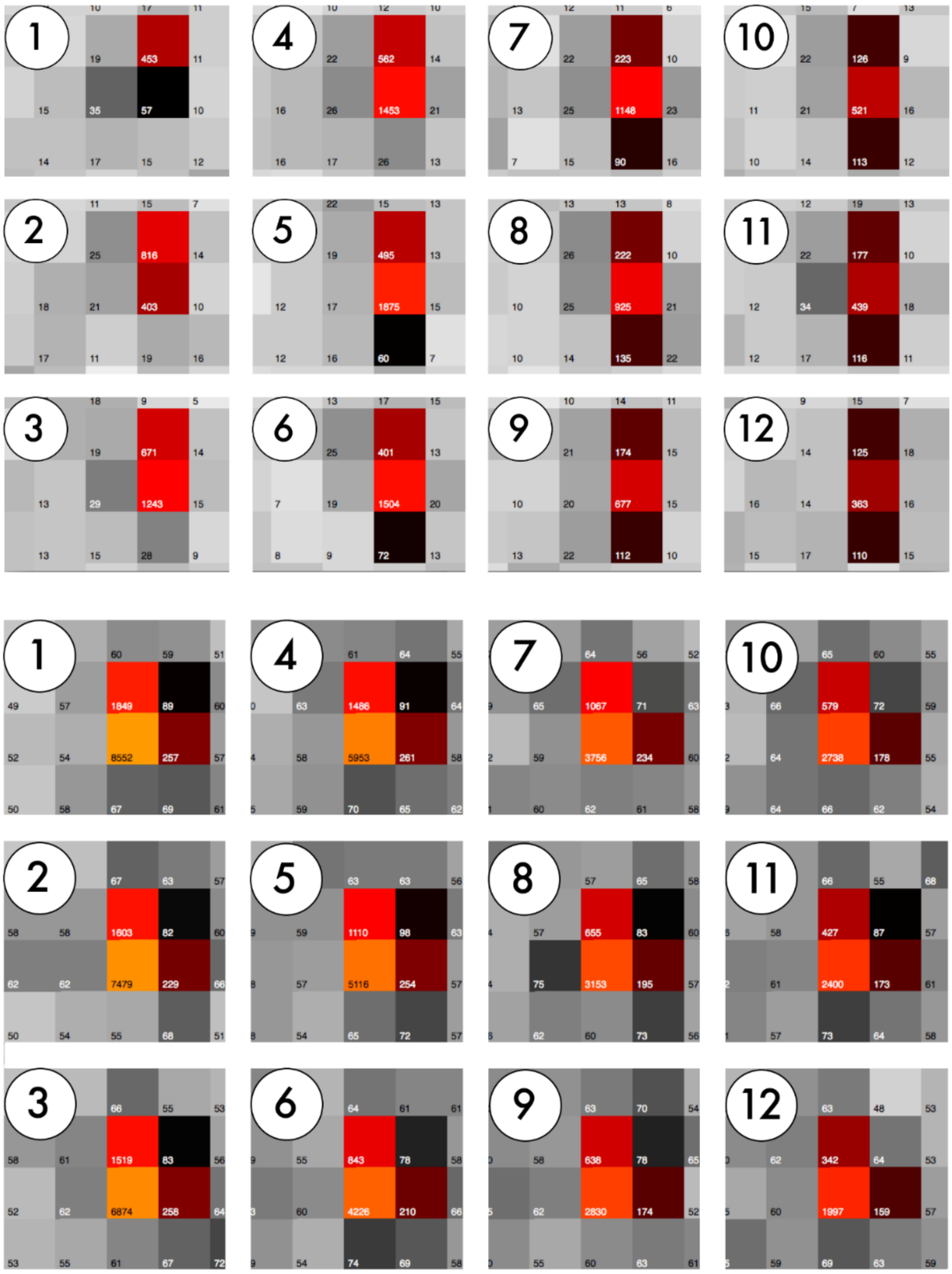

Figure S7 Examples of the evolution of peak intensity with dose, as indicated by detector pixel counts, for thaumatin crystals with a nominal incident dose rate of 36 MGy/s, a detector frame rate of 500 Hz, and a detector counting time of 1 ms. Large numbers in circles refer to detector frame numbers, and small numbers in the lower left corner of each pixel gives the photon count. The dose per frame is 71 kGy. (Top) A peak measured at T=260 K, which initially brightens with increasing dose before dimming at larger doses. (Bottom). A peak measured at T=300 K that monotonically dims with increasing dose.

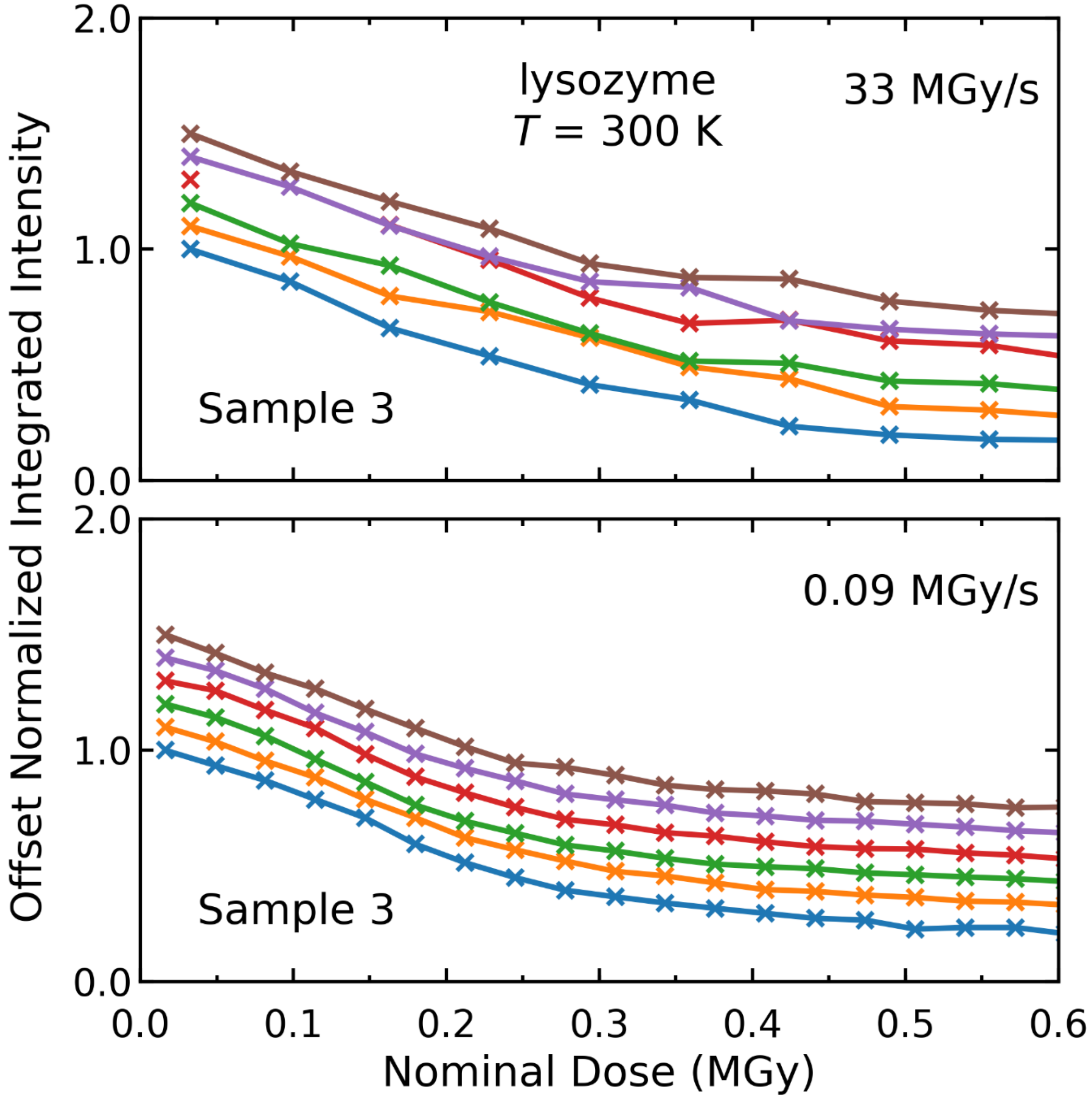

Figure S8 Comparison of integrated intensity versus dose data acquired at multiple positions on the same lysozyme sample at T=300 K, using (top) a 33 MGy/s dose rate and 500 Hz detector frame rate (0.066 MGy dose steps) and (bottom) using a 0.04 MGy/s dose rate and 1.2 Hz frame rate (0.033 MGy dose steps). At large dose rates, transient upward fluctuations in integrated intensity are observed for doses comparable to or larger than the half-dose.

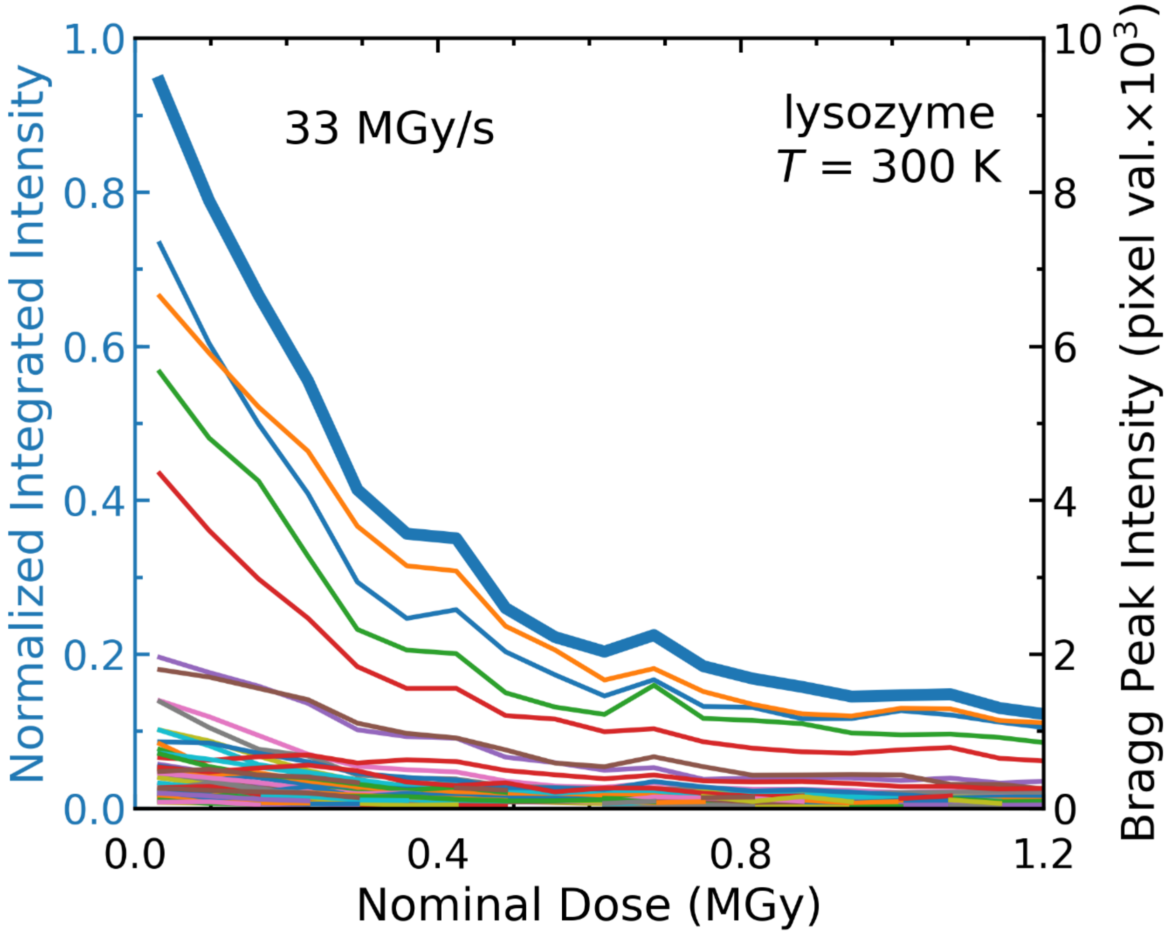

Figure S9 Plots of the integrated intensity (heavy blue line) and the intensity of individual Bragg peaks vs dose for the lysozyme crystal of Fig. S8 at T=300 K, for a dose rate of 33 MGy/s and a 500 Hz detector frame rate. Upward jumps in the integrated intensity correspond to jumps in the individual Bragg peak intensities, and have a duration of 2-4 ms.

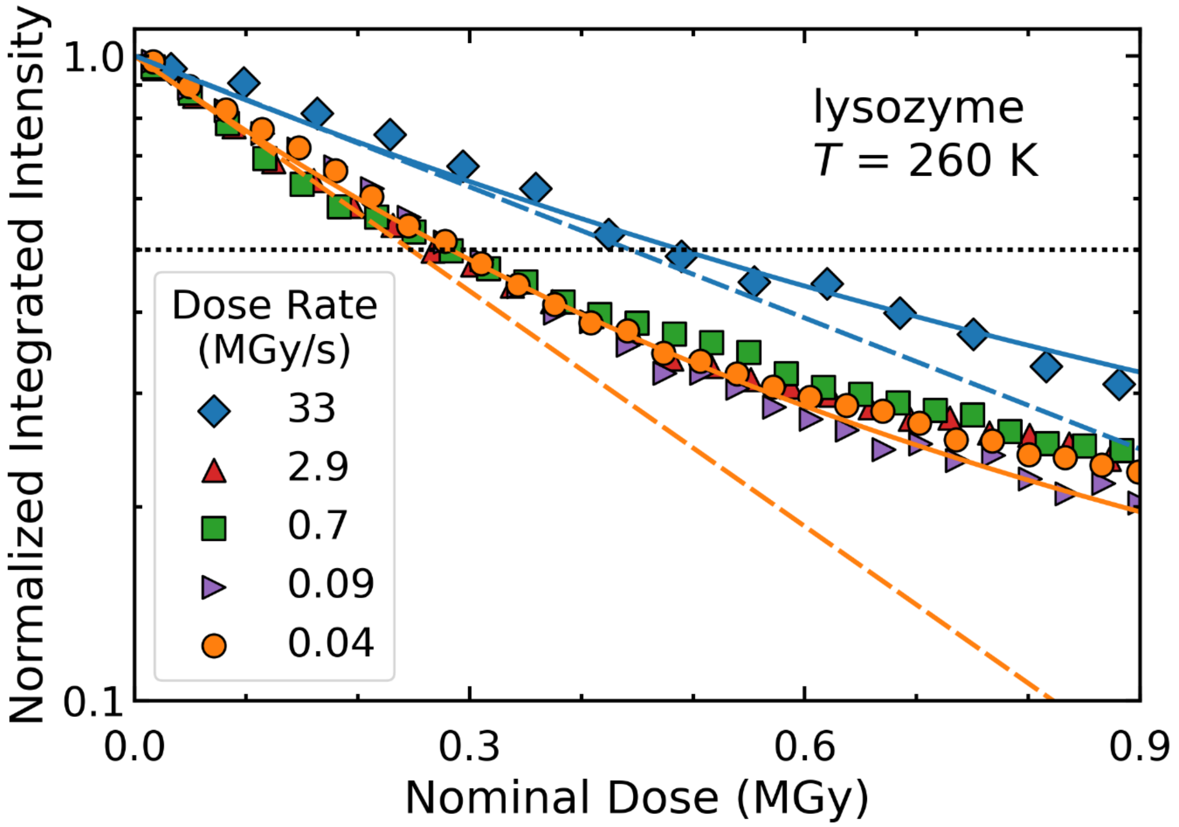

Figure S10 Representative semi-log plots of the integrated intensity in diffraction peaks versus dose at several dose rates, acquired from a single lysozyme crystal at 260 K, as in Figure S5, but as calculated by integrating the normalized peak intensities *I*/σ determined using XDS instead of the absolute intensities generated by DISTL.

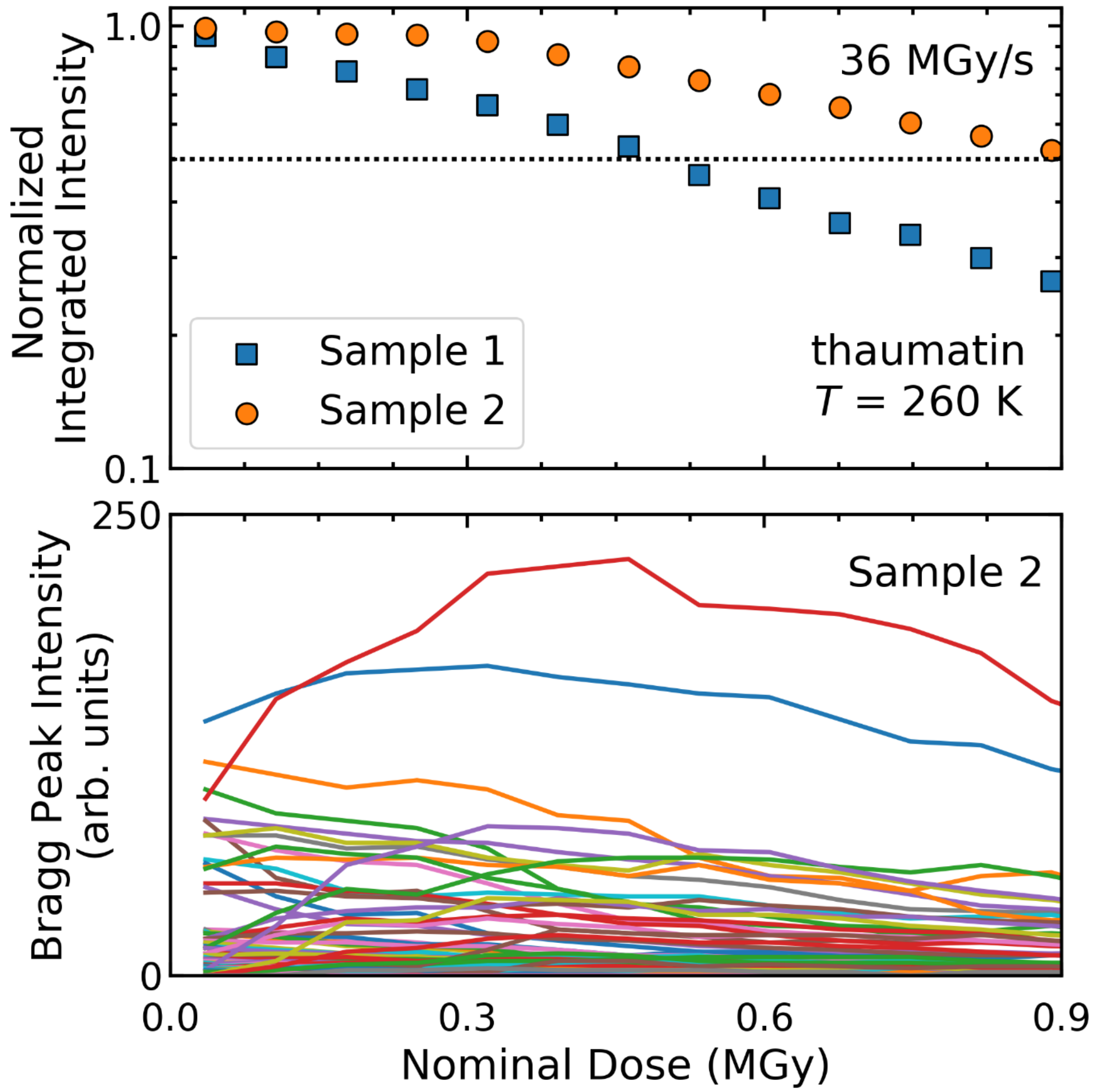

Figure S11 (Top) Representative integrated frame intensity vs dose curves for two thaumatin crystals at 260 K for a nominal dose rate of 36 MGy/s, as in Fig. S6 (top) but calculated using normalized individual peak intensities I/σ determined using XDS instead of the absolute background-subtracted intensities generated by DISTL. (Bottom) XDS-generated normalized peak intensities *I*/*σ* corresponding to the data in Fig. S6 (bottom).
